# Multi-omics characterization of esophageal squamous cell carcinoma identifies molecular subtypes and therapeutic targets

**DOI:** 10.1172/jci.insight.171916

**Published:** 2024-04-23

**Authors:** Dengyun Zhao, Yaping Guo, Huifang Wei, Xuechao Jia, Yafei Zhi, Guiliang He, Wenna Nie, Limeng Huang, Penglei Wang, Kyle Vaughn Laster, Zhicai Liu, Jinwu Wang, Mee-Hyun Lee, Zigang Dong, Kangdong Liu

**Affiliations:** 1 Department of Pathophysiology, School of Basic Medical Sciences, Academy of Medical Sciences, Zhengzhou University, Zhengzhou, Henan, China.; 2 China-US (Henan) Hormel Cancer Institute, Zhengzhou, Henan, China.; 3 Tianjian Laboratory of Advanced Biomedical Sciences, Zhengzhou, Henan, China.; 4 The Collaborative Innovation Center of Henan Province for Cancer Chemoprevention, Zhengzhou, Henan, China.; 5 State Key Laboratory of Esophageal Cancer Prevention and Treatment, Zhengzhou University, Zhengzhou, Henan, China.; 6 Linzhou Cancer Hospital, Anyang, Henan, China.; 7 College of Korean Medicine, Dongshin University, Naju, Jeonnam, Republic of Korea.; 8 Provincial Cooperative Innovation Center for Cancer Chemoprevention, Zhengzhou University, Zhengzhou, Henan, China.

**Keywords:** Oncology, Therapeutics, Cancer, Protein kinases, Proteomics

## Abstract

Esophageal squamous cell carcinoma (ESCC) is the predominant form of esophageal cancer and is characterized by an unfavorable prognosis. To elucidate the distinct molecular alterations in ESCC and investigate therapeutic targets, we performed a comprehensive analysis of transcriptomics, proteomics, and phosphoproteomics data derived from 60 paired treatment-naive ESCC and adjacent nontumor tissue samples. Additionally, we conducted a correlation analysis to describe the regulatory relationship between transcriptomic and proteomic processes, revealing alterations in key metabolic pathways. Unsupervised clustering analysis of the proteomics data stratified patients with ESCC into 3 subtypes with different molecular characteristics and clinical outcomes. Notably, subtype III exhibited the worst prognosis and enrichment in proteins associated with malignant processes, including glycolysis and DNA repair pathways. Furthermore, translocase of inner mitochondrial membrane domain containing 1 (TIMMDC1) was validated as a potential prognostic molecule for ESCC. Moreover, integrated kinase-substrate network analysis using the phosphoproteome nominated candidate kinases as potential targets. In vitro and in vivo experiments further confirmed casein kinase II subunit α (CSNK2A1) as a potential kinase target for ESCC. These underlying data represent a valuable resource for researchers that may provide better insights into the biology and treatment of ESCC.

## Abstract 2

Multi-omics analysis of esophageal squamous cell carcinoma identifies distinct molecular subtypes associated with prognosis and potential kinase targets.

## Graphical abstract

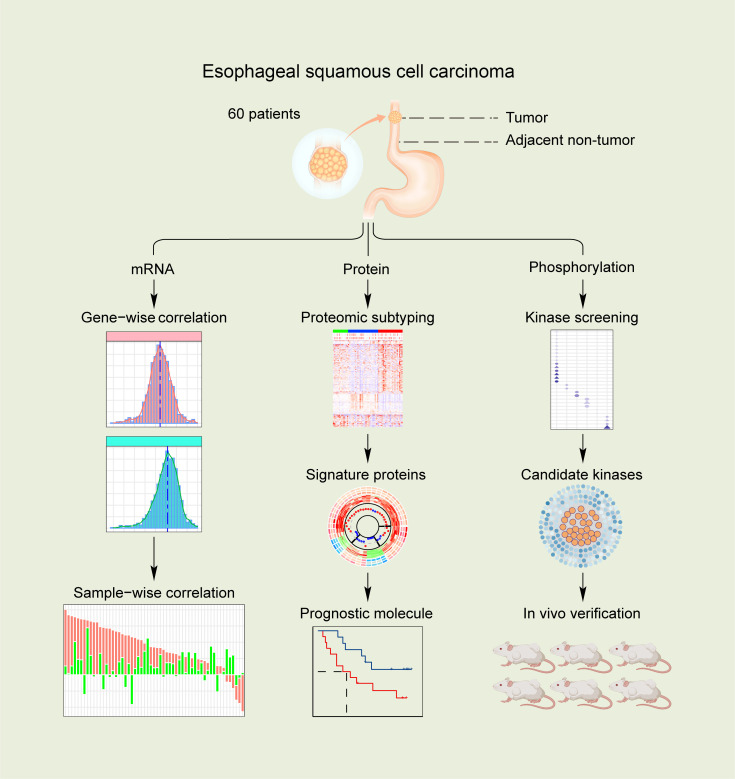

## Introduction

Esophageal cancer is a highly aggressive malignancy and is the sixth most common cause of cancer-related mortality worldwide (1, 2). Esophageal squamous cell carcinoma (ESCC) and esophageal adenocarcinoma (EAC) are the 2 main histopathological subtypes of esophageal cancer, among which ESCC accounts for approximately 90% of the diagnosed cases (3). Current therapeutic options for ESCC include endoscopic therapy, surgery, chemotherapy, radiotherapy, and comprehensive treatment. Several studies (4–10) have demonstrated that various inhibitors targeting VEGFR (e.g., apatinib and anlotinib), HER-2 (e.g., lapatinib), and PD-1/PD-L1 (e.g., pembrolizumab, camrelizumab, and toripalimab) can improve the survival rates of patients with ESCC. Currently, pembrolizumab, lapatinib, and anlotinib are approved for the first- or second-line treatment of ESCC (6–8). Despite advancements in treatment strategies, patient clinical outcomes remain unsatisfactory, primarily due to the heterogeneity of individual patients, lack of early specific symptoms, and absence of effective screening strategies, resulting in diagnosis commonly occurring at advanced stages.

In the past decade, large-scale sequencing efforts have been made to investigate the underlying molecular alterations in ESCC. For instance, comprehensive genomic studies (11–13) have revealed that TP53, RB1, CDKN2A, NOTCH1, and FAT1 are the most frequently mutated genes, and this understanding has advanced our knowledge of the pathogenic mechanisms involved in ESCC. Currently, multidimensional ‘‘omics’’ strategies encompassing proteomic and phosphoproteomic profiling in conjunction with other omics analyses have elucidated molecular subtypes and signaling pathways, revealing potential therapeutic targets for diverse cancer types (14–19). In a recent study (20), ESCC was classified into 2 different molecular subtypes (S1 and S2) based on proteomics data, and 3 potentially effective drugs were validated for the treatment of patients with the S2 subtype. However, a comprehensive phosphoproteomics analysis was not conducted due to the small sample size (*n* = 31 tissue pairs). In another study (21) involving 94 tumor and 24 nontumor tissue samples, 3 distinct ESCC subtypes (subtype I [S-I], S-II, and S-III) were identified, and the therapeutic potential of TG003, a CLK inhibitor, was confirmed specifically for patients with S-III. These investigations have enhanced our understanding of ESCC pathogenesis and potential therapeutic strategies for ESCC. However, ESCC patient-paired cohorts with more extensive survival data are needed to further elucidate the molecular alterations contributing to ESCC initiation and development.

In this study, we performed a comprehensive transcriptomics, proteomics, and phosphoproteomics analysis of treatment-naive ESCC and paired adjacent nontumor tissue samples obtained from 60 patients over a 7-year follow-up period. A correlation analysis between transcriptomics and proteomics data was conducted, revealing alterations in key metabolic pathways. Unsupervised clustering of the proteome identified 3 subtypes (S-I, S-II, and S-III) associated with different clinical and molecular features. Moreover, integrated kinase substrate network analysis based on the phosphoproteome predicted potential kinase targets, which were further verified in experiments. In summary, our study provides complementary information on ESCC that may contribute to a better understanding of this disease and the development of more effective treatment strategies.

## Results

### Multi-omic landscape of ESCC samples.

**Figure 1:**
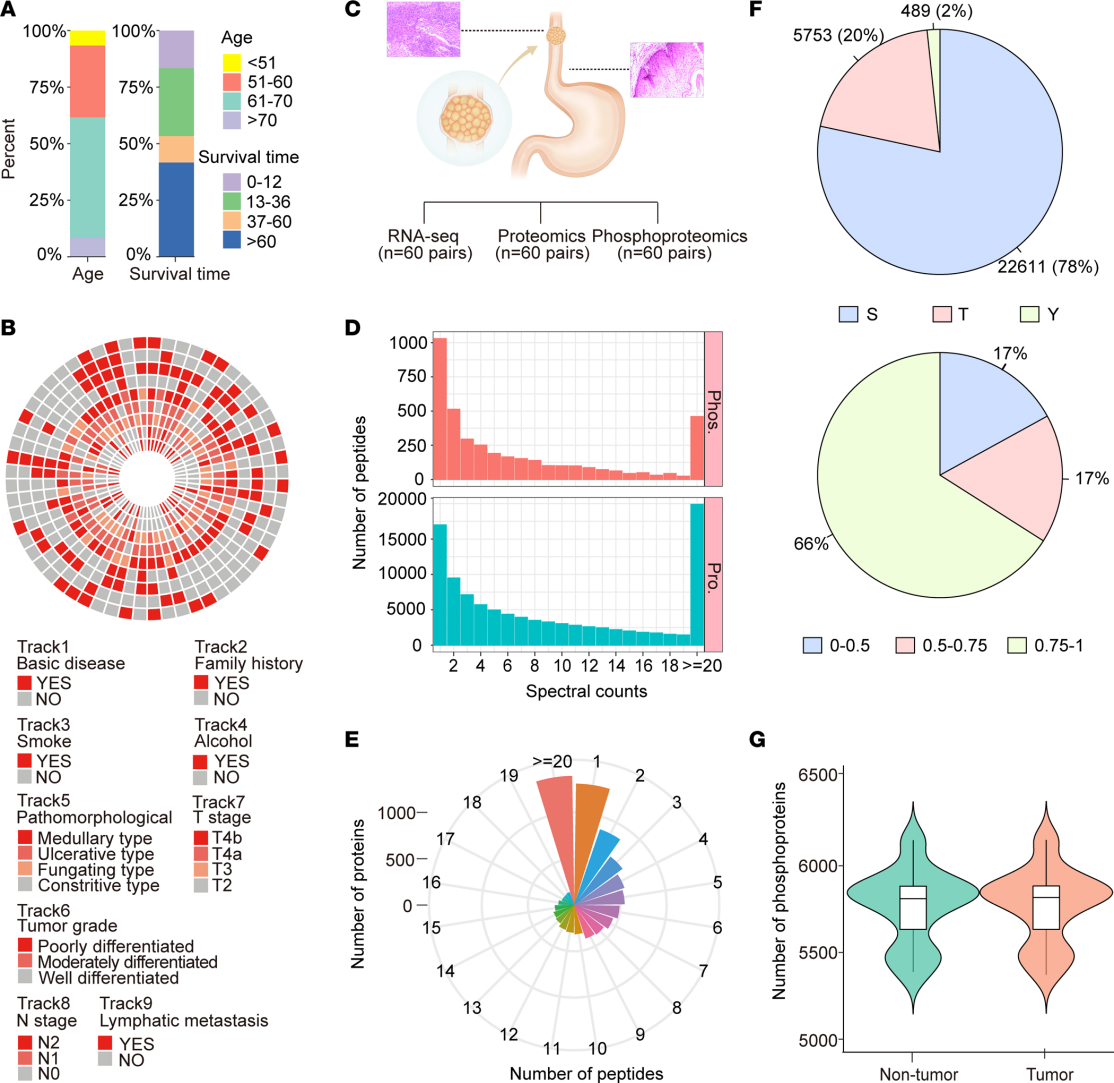
Summary of RNA-Seq, proteomics, and phosphoproteomics analysis in ESCC. (**A**) Stacked histograms depicting the distribution of age and survival time of 60 patients. Statistical unit for age: year; statistical unit for survival time: month. (**B**) Circular heatmap illustrating the distribution of clinical information among 60 patients with ESCC. Each axis represents an individual patient, and each track represents corresponding patient information. (**C**) Schematic illustrating the flowchart of this study. (**D**) Histograms illustrating the raw LC-MS/MS spectral counts distribution of peptides and phosphopeptides identified from proteomics and phosphoproteomics data, respectively. Pro., proteomics; Phos., phosphoproteomics. (**E**) Circular bar plot displaying the peptide number distribution identified from proteomics data. (**F**) Pie charts presenting the composition of phosphorylated residue type (p-Ser, p-Thr, and p-Tyr) (top), as well as assigned localization probability (bottom) for all detected phosphosites. (**G**) Violin plot illustrating the number of quantified phosphoproteins in paired tumor and adjacent nontumor tissues.

In the present study, paired tumor and adjacent nontumor samples were collected from 60 treatment-naive Chinese patients with ESCC. The samples represented diverse clinical and pathological characteristics including age, sex, and tumor grade and stage. Notably, the 7-year follow-up information was also recorded (Figure 1, A and B, and Supplemental Table 1; supplemental material available online with this article; https://doi.org/10.1172/jci.insight.171916DS1). For all 60 paired samples, mRNA-Seq (RNA-Seq) and the tandem mass tag–based (TMT-based) isobaric labeling strategy were employed for precise relative quantification of global mRNAs, proteins, and phosphosites (Figure 1C and Supplemental Figure 1, A and B). At the transcriptional level, a total of 19,615 RNAs were quantified (Supplemental Figure 1C and Supplemental Table 2). TMT-based proteomics and phosphoproteomics analyses revealed a total of 100,898 nonphosphorylated peptides and 28,853 phosphopeptides, the majority of which exhibited 2 or more spectral counts (Figure 1D). Subsequently, all nonphosphorylated peptides were aligned to their corresponding protein sequences, resulting in mapping to 9,261 proteins, of which 8,224 were quantifiable and only a minority were mapped to 1 peptide (Figure 1E). Among the 28,853 identified phosphopeptides, there were 22,611 phosphorylated Ser (p-Ser) (78%), 5,753 p-Thr (20%), and 489 p-Tyr (2%) peptides, most of which had a localization probability > 0.75 (Figure 1F). In total, we identified 18,944 phosphosites on 5,213 phosphoproteins with a confident site localization score (localization probability > 0.75) (Figure 1G). Principal component analysis (PCA) based on the proteomics data revealed no discernible batch effect among the TMT plexes (Supplemental Figure 1D), and the relative quantification information is illustrated in Supplemental Figure 1E. The ESTIMATE analysis of RNA-Seq data revealed a higher immune cell infiltration in adjacent nontumor samples (Supplemental Table 2). The results presented herein demonstrate the high quality of both the samples and the data.

### Correlation analysis of transcriptomics and proteomics data.

**Figure 2:**
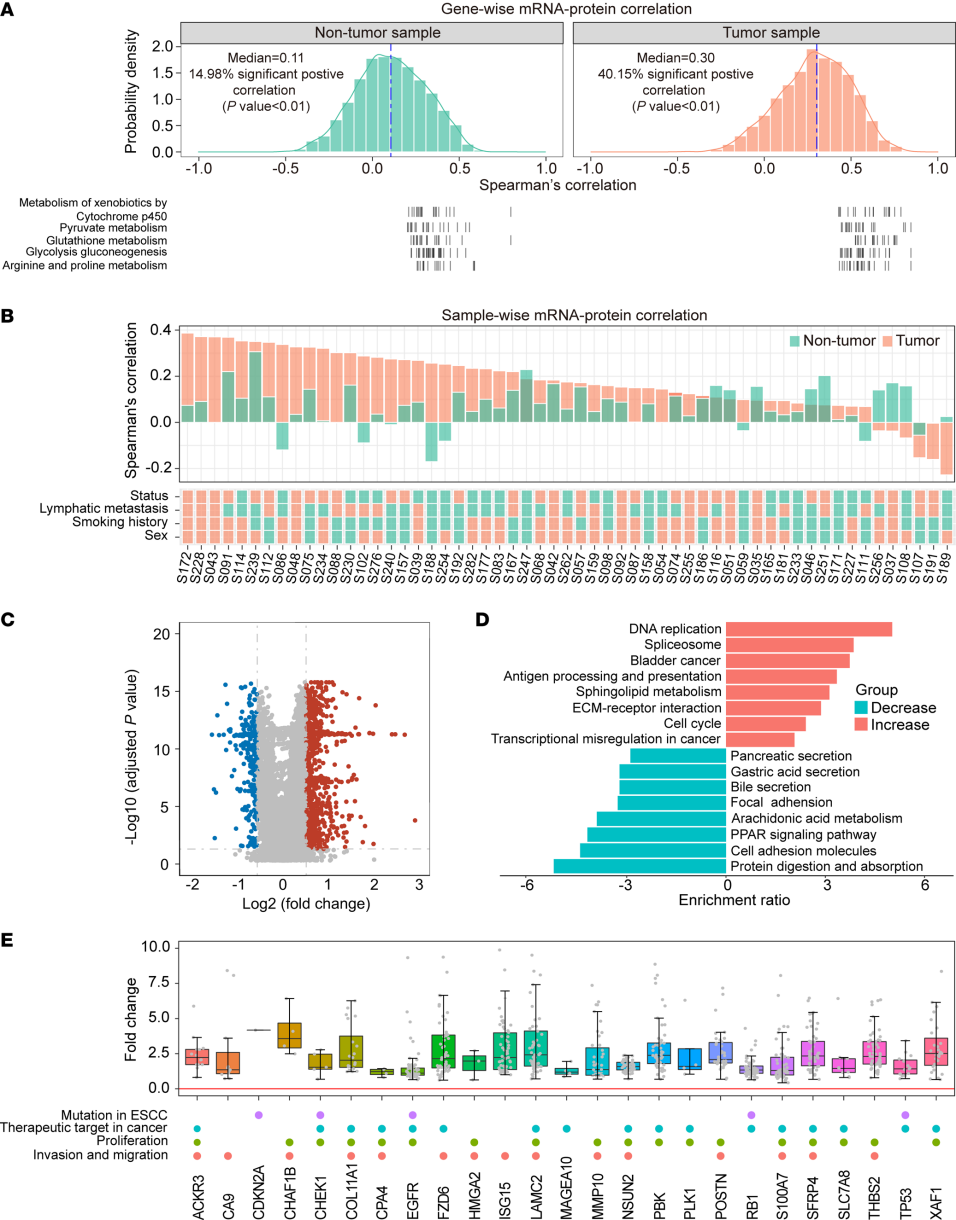
ESCC proteomic profiling in tumor and adjacent nontumor samples. (**A**) Histogram illustrating the gene-wise correlations of mRNA and protein expression in adjacent nontumor (left, *n* = 60) and tumor tissues (right, *n* = 57). Green represents adjacent nontumor tissue, and red represents tumor tissue. (**B**) Histogram illustrating the sample-wise correlations of adjacent nontumor (*n* = 57) and tumor tissues (*n* = 57). Green represents adjacent nontumor tissue, and red represents tumor tissue. Living status, lymphatic metastasis, smoking history, and sex of each patient are listed directly below the histogram. Green represents living patients, and red represents deceased patients; green represents no lymphatic metastasis, and red represents lymphatic metastasis; green represents nonsmoking history, and red represents a smoking history; and green represents female patients, and red represents male patients. (**C**) Volcano plot indicating differential proteins between tumor and adjacent nontumor tissues. Red dots represent upregulated proteins in tumor tissues compared with adjacent nontumor tissues, while blue dots represent downregulated proteins. Gray dots represent proteins with no significant difference between tissues. (**D**) Enriched KEGG pathways of upregulated and downregulated proteins in tumor tissues compared with adjacent nontumor tissues. (**E**) Histogram illustrating differential expression of representative proteins along with their mutation status, target potential, and cancer-related phenotypes such as proliferation, invasion, and migration.

To characterize the relationship between transcriptomic and proteomic processes in ESCC, gene-wise (intersample) and sample-wise (intrasample) correlations of 5,676 mRNA-protein pairs from 57 tumor and 60 adjacent nontumor tissues were calculated. The median correlation coefficient for adjacent nontumor tissues was 0.11, while that for tumor tissues was 0.30 (Figure 2A). The adjacent nontumor and tumor tissues of ESCC exhibited significant positive Spearman correlation coefficients of 14.98% and 40.15%, respectively, for mRNA-protein pairs (Spearman’s correlation, *P* < 0.01). The enrichment analysis indicated alterations in key metabolic pathways, including xenobiotics by cytochrome p450 metabolism, pyruvate metabolism, glutathione metabolism, and glycolysis gluconeogenesis (Figure 2A). Additionally, the sample-wise mRNA-protein correlation analysis revealed a higher correlation value in tumor tissues than in adjacent nontumor tissues (Figure 2B and Supplemental Table 3).

### Proteomic features of tumor tissues compared with adjacent nontumor tissues.

Given that proteins serve as crucial functional executors within cells, we performed an in-depth proteomics analysis. The proteome-based correlation analysis demonstrated decreased correlations in tumors compared with adjacent nontumors, suggesting high individual tumor heterogeneity in ESCC (Supplemental Figure 2A). Moreover, we identified 751 upregulated and 274 downregulated proteins in tumor tissues compared with adjacent nontumor tissues among the 8,224 quantified proteins (Figure 2C and Supplemental Table 2). KEGG pathway enrichment analysis revealed that the upregulated proteins were enriched in DNA replication, spliceosome, and antigen processing and presentation, while the downregulated proteins were enriched in protein digestion and absorption, cell adhesion, PPAR signaling pathway, and arachidonic acid metabolism pathway (Figure 2D). In addition, our enrichment analysis based on cancer hallmark gene sets from the Molecular Signatures Database (MsigDB) revealed substantial involvement of these dysregulated proteins in malignancy-associated pathways, including epithelial-mesenchymal transition (EMT), E2F targets, G2/M checkpoint, and mTORC1 signaling pathway (Supplemental Figure 2B).

We also identified a series of proteins associated with cancer development that have been previously reported. Specifically, the protein levels of genes frequently mutated in ESCC — including CDKN2A, CHEK1, EGFR, RB1, and TP53 — were found to be upregulated (12). Other notable proteins, such as ACKR3, COL11A1, CPA4, and FZD6, were previously reported (22–25) as potential therapeutic targets in cancer. Moreover, previous studies (26–32) have implicated ACKR3, CHAF1B, CHEK1, and COL11A1 as being involved in cancer cell proliferation, while CA9, CHAF1B, and COL11A1 play roles in cancer invasion and migration (Figure 2E). These findings provide valuable insights for understanding the biology of ESCC. In addition, subcellular localization analysis revealed that the differentially regulated proteins were predominantly localized in the nucleus, cytoplasm, and plasma membrane (Supplemental Figure 2C).

### Protein abundance-based subtyping of ESCC.

**Figure 3:**
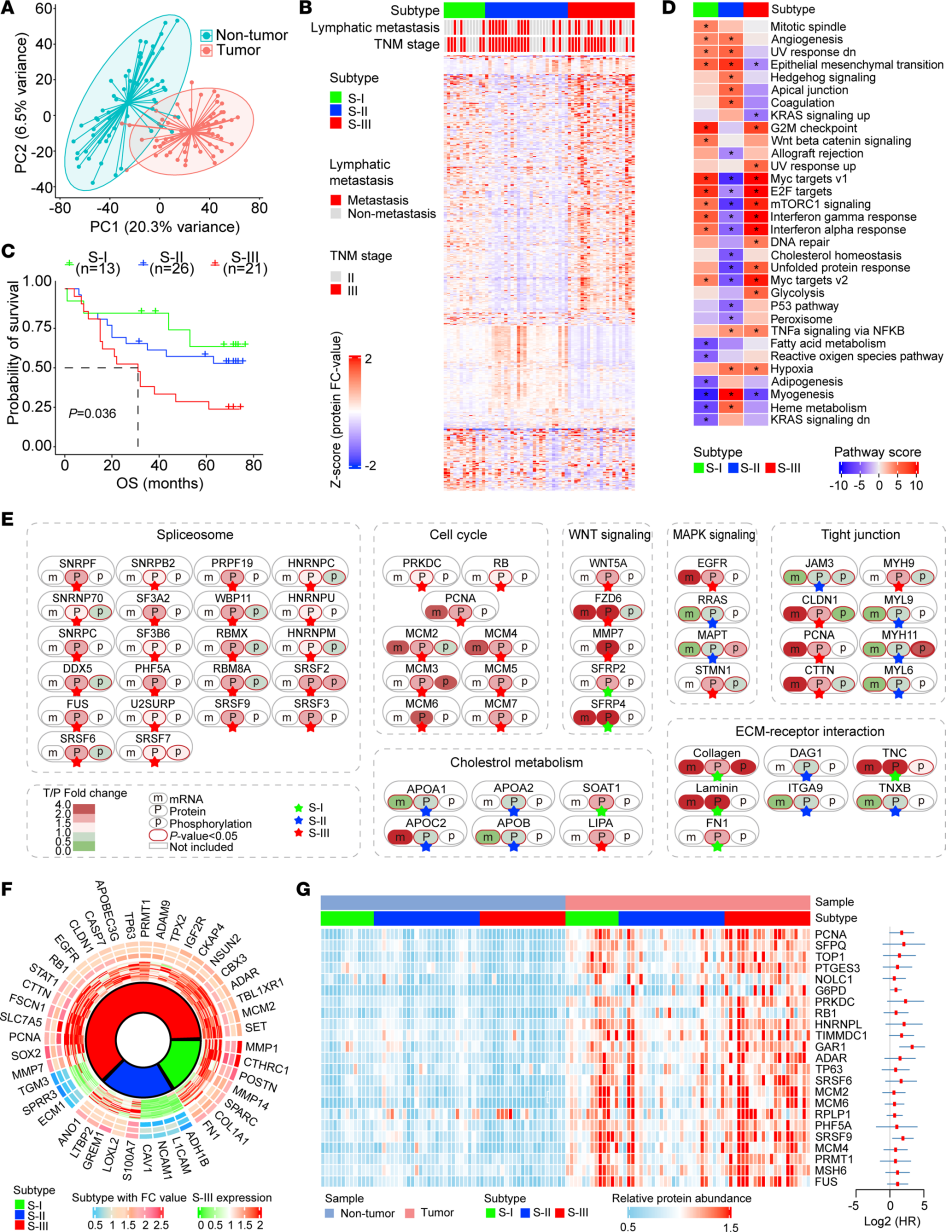
Molecular subtyping of ESCC. (**A**) PCA of protein expression in tumor and adjacent nontumor samples. The red oval represents tumor tissue, and the green oval represents adjacent nontumor tissue. (**B**) Heatmap shows 3 consensus clusters based on the proteomics data, with their associations with clinical information such as lymphatic metastasis and TNM stages presented in the middle panel. (**C**) Kaplan-Meier plot comparing survival probability of patients in S-I to S-III. (**D**) Heatmap elucidating the major signaling pathways enriched in S-I to S-III. (**E**) Integrative molecular network analysis reveals the expression of mRNA, protein, and phosphoprotein for representative molecular of S-I to S-III. (**F**) Circular heatmap displaying representative molecular characteristics of S-I to S-III. (**G**) Expression levels and HRs for selected proteins in S-III.

PCA based on the proteomics data indicated a clear boundary between tumor and adjacent nontumor tissues (Figure 3A). By employing unsupervised consensus clustering analysis, we identified 3 subtypes (S-I, S-II, and S-III) based on the differentially expressed proteins (Figure 3B and Supplemental Figure 3, A and B). To explore potential associations between clinicopathological characteristics and these 3 subtypes, we evaluated TNM stage and occurrence of lymphatic metastasis in each patient based on subtype. Interestingly, the incidence of lymphatic metastasis was higher in S-II (46.2%) and S-III (47.6%) compared with S-I (23.1%). Additionally, a greater proportion of patients diagnosed with stage III were observed in S-II (61.5%) and S-III (71.4%) compared with S-I (53.8%) (Figure 3B). Further analysis demonstrated that patients with S-I had the best prognosis, whereas patients with S-III had the worst prognosis (Log-rank test, *P* = 0.036) (Figure 3C). Enrichment analysis demonstrated that metabolism-related processes such as fatty acid metabolism, adipogenesis, and reactive oxygen species were exclusively dysregulated in S-I tumors, demonstrating the metabolic disorder characteristics of these tumors. The Hedgehog signaling pathway, apical junction pathway, and coagulation pathway were found to be enriched within tumors classified as S-II. In contrast, the glycolysis pathway, DNA repair process, Myc, and cell cycle pathways were enriched within tumors categorized as S-III, suggesting the proliferative characteristics of these tumors (Figure 3D).

Moreover, integrative molecular network analysis further revealed that, in contrast to S-I and S-II, S-III exhibited aggressive characteristics, including the upregulation of proteins involved in pathways associated with spliceosome (such as SNRPF, PRPF19, and SRSF6) and cell cycle (such as PCNA, MCM2, and MCM4) (Figure 3E and Supplemental Table 4). These findings are consistent with a previous study (20), which suggested that cell cycle and spliceosome-related proteins play crucial roles in ESCC tumorigenesis. Additionally, previous studies reported (12, 33–38) that ESCC-associated proteins, including PCNA, SLC7A5, CTTN, RB1, EGFR, TP63, and PRMT1, exhibited increased expression in S-III; among these proteins, SLC7A5 and EGFR were FDA-approved drug targets for cancer treatment (Figure 3F). Cox regression analysis was subsequently performed to assess the potential prognostic molecules in S-III. A series of proteins, including GAR1, PRKDC, SRSF9, SRSF6, SFPQ, RB1, TP63, and translocase of inner mitochondrial membrane domain containing 1 (TIMMDC1), were associated with a high prognosis risk score; among those proteins, PRKDC, RB1, and TP63 have been previously reported (39) in ESCC (Figure 3G). Based on 4 criteria (described in Methods), we nominated TIMMDC1 (Wilcoxon test, *P* = 3 × 10^–13^; Log-rank test, *P* = 0.041) as a potential prognostic molecule for further investigation (Supplemental Figure 3, C and D).

### Upregulated TIMMDC1 predicts poor prognosis and promotes ESCC development.

**Figure 4:**
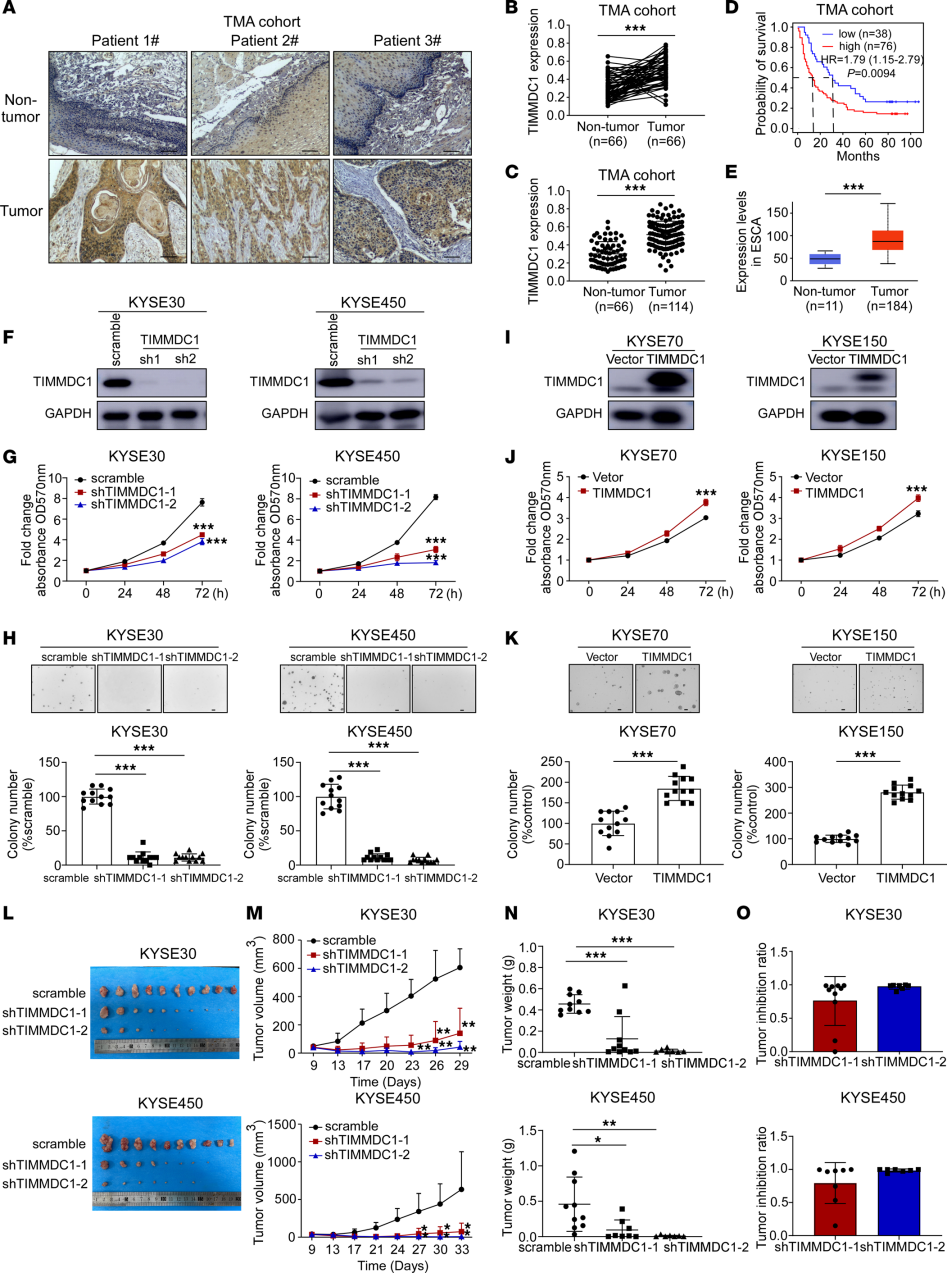
Elevated expression of TIMMDC1 promotes ESCC growth and is associated with poor prognosis. (**A**) Representative IHC staining images of a human ESCC TMA using a specific antibody for TIMMDC1 are shown. Scale bar: 100 μm. (**B** and **C**) Protein levels of TIMMDC1 were analyzed in an ESCC TMA, including paired tumor and adjacent nontumor tissues of 66 patients (**B**) and tumors without paired nontumor tissues for of patients (**C**). (**D**) Kaplan-Meier plot comparing the survival probability of patients with high/low TIMMDC1 expression. (**E**) Transcript expression level of TIMMDC1 was determined in the TCGA-ESCA patient cohort. (**F**) TIMMDC1 knockdown efficiency was assessed by Western blot. (**G** and **H**) Cell proliferation was measured by MTT (*n* = 6 for each group) (**G**) and soft agar (*n* = 12 for each group) assays (**H**) after knockdown of TIMMDC1. Scale bar: 200 μm. (**I**) TIMMDC1 overexpression efficiency was assessed by Western blot. (**J** and **K**) Cell proliferation was measured by MTT (*n* = 6 for each group) (**J**) and soft agar (*n* = 12 for each group) assays (**K**) after overexpression of TIMMDC1. Scale bar: 200 μm. (**L**–**O**) KYSE30 and KYSE450 cells stably expressing scramble or shTIMMDC1 were s.c. injected into the right flank of each mouse (KYSE30: scramble, *n* = 10; sh1, *n* = 10; sh2, *n* = 7; KYSE450: scramble, *n* = 10; sh1, *n* = 8; sh2, *n* = 7). Tumors were excised at the end of the experiment. (**L**) Images of xenograft tumors. (**M**) Xenograft tumor growth curves in mice. (**N**) Weights of xenograft tumors. (**O**) Tumor inhibition ratios of TIMMDC1 knockdown. In all statistical plots, data were expressed as the mean ± SD. Two-tailed Student’s *t* test (**B**, **C**, **E**, **J**, and **K**) and 1-way ANOVA (**G**, **H**, **M**, and **N**) were used to determine statistical significance. *P < 0.05, **P < 0.01, ***P < 0.001. Representative results from at least 3 independent biological replicates (**F**–**K**) are shown.

TIMMDC1, a crucial factor for the assembly of OXPHOS complex I, plays an important role in cellular energy homeostasis under stress conditions (40, 41). A previous study (42) demonstrated that increased levels of TIMMDC1 can promote gastric cancer proliferation. To explore the role of TIMMDC1 in ESCC, we evaluated its expression in a human ESCC tissue microarray (TMA) cohort by IHC staining. Consistent with our proteomics analysis shown in Supplemental Figure 3C, we observed significant upregulation of TIMMDC1 in tumor tissues compared with nontumor tissues (Figure 4, A–C). Furthermore, a Kaplan-Meier analysis demonstrated that patients with elevated TIMMDC1 expression had significantly shorter cancer-related survival times than those with low TIMMDC1 expression in the TMA cohort (Log-rank test, *P* = 0.0094) (Figure 4D). Moreover, analysis of RNA-Seq data from the TCGA-ESCA cohort revealed significantly increased TIMMDC1 transcript levels in tumor tissues (*P* < 0.0001) (Figure 4E). TIMMDC1 protein levels were determined by Western blotting in paired ESCC and nontumor tissues as well as in different ESCC cell lines. Elevated TIMMDC1 protein levels were observed in most tumor tissues compared with nontumor tissues (Supplemental Figure 4A). In addition, TIMMDC1 was upregulated in ESCC cell lines compared with the human immortalized normal esophageal epithelial cell line (Shantou human embryonic esophageal cell line [SHEE]) (Supplemental Figure 4B). These findings suggest that TIMMDC1 is a potential prognostic molecule in ESCC.

To investigate the molecular function of TIMMDC1 in ESCC, 2 specific short hairpin TIMMDC1 (shTIMMDC1) sequences were designed to knock down TIMMDC1 in the KYSE30 and KYSE450 cell lines, which express relatively high levels of TIMMDC1 (Supplemental Figure 4B). TIMMDC1 protein levels were decreased in shTIMMDC1 cells compared with scramble cells (Figure 4F). Subsequently, MTT, soft agar, and colony formation assays were performed to assess the effect of TIMMDC1 knockdown on ESCC cell proliferation and colony formation. Our findings demonstrate that knockdown of TIMMDC1 resulted in decreased cell proliferation and suppressed colony formation (Figure 4, G and H, and Supplemental Figure 4, C and D). Furthermore, we investigated whether the overexpression of TIMMDC1 could enhance cell proliferation and colony formation in KYSE70 and KYSE150 cells with relatively low levels of TIMMDC1 expression (Supplemental Figure 4B and Figure 4I). As expected, TIMMDC1 overexpression promoted cell proliferation and colony formation (Figure 4, J and K, and Supplemental Figure 4, E and F). To evaluate the effect of TIMMDC1 on tumor growth in vivo, a cell-derived xenograft (CDX) model was established. Our results demonstrate that TIMMDC1 knockdown inhibited tumor growth and reduced tumor weights compared with those in the scramble group (Figure 4, L–O). In summary, these results provide evidence to support the potential of TIMMDC1 as a promising prognostic and therapeutic molecule for ESCC.

### Identification of potentially activated protein kinases.

In total, 3,368 phosphoproteins were quantified after normalization of the proteomics and phosphoproteomics data. Among the quantified proteins, we identified 1,113 phosphosites on 689 proteins that were upregulated in tumor tissues; on the other hand, 606 phosphosites on 441 proteins were downregulated (Supplemental Table 2 and Supplemental Figure 5A). Enrichment analysis based on differentially expressed phosphoproteins revealed that the upregulated phosphoproteins showed enrichment mainly in pathways related to Notch signaling, RNA transport, spliceosome, adhesion junction, and cell cycle. Moreover, the downregulated phosphoproteins showed enrichment mainly in pathways associated with spliceosome, tight junction, adhesion junction, and regulation of actin cytoskeleton (Supplemental Figure 5B and Supplemental Table 5). To elucidate the disparities in phosphoproteomic profiles among the patients’ proteomic subtypes, we integrated the phosphoproteomics data into our proteomic subtyping analysis. We observed substantial variations in the abundance of quantified phosphosites across the 3 subtypes (Supplemental Figure 5C). For instance, TP53BP1 is involved in DNA double-strand break repair, and our results reveal that TP53BP1 S1678 exhibited the highest level in S-III (S-I vs. S-II, 2-tailed Student’s *t* test, *P* = 0.0062; S-I vs. S-III, 2-tailed Student’s *t* test, *P* = 0.05; S-II vs. S- III, 2-tailed Student’s *t* test, *P* = 7 × 10^–5^). This finding aligns with our protein subtype analysis, which identified DNA repair as one of the pathways represented in S-III. Moreover, a previous study (43) reported that CDK1-dependent phosphorylation of TP53BP1 at S1678 impairs its binding to importin during mitosis. Additionally, it has been reported (44) that CK2-dependent phosphorylation of PAK1 at S223 necessitates its catalytically active monomeric form. Our analysis revealed that PAK1 S223 exhibited the highest level in S-III and the lowest in S-I (S-I vs. S-II, *t* test, *P* = 0.28; S-I vs. S-III, 2-tailed Student’s *t* test, *P* = 4.6 × 10^–4^; S-II vs. S-III, 2-tailed Student’s *t* test, *P* = 0.0021). Furthermore, we discovered several phosphoproteins associated with the 3 subtypes, including MAP2, MABPP1A, and PGM5 (Supplemental Figure 5C).

**Figure 5:**
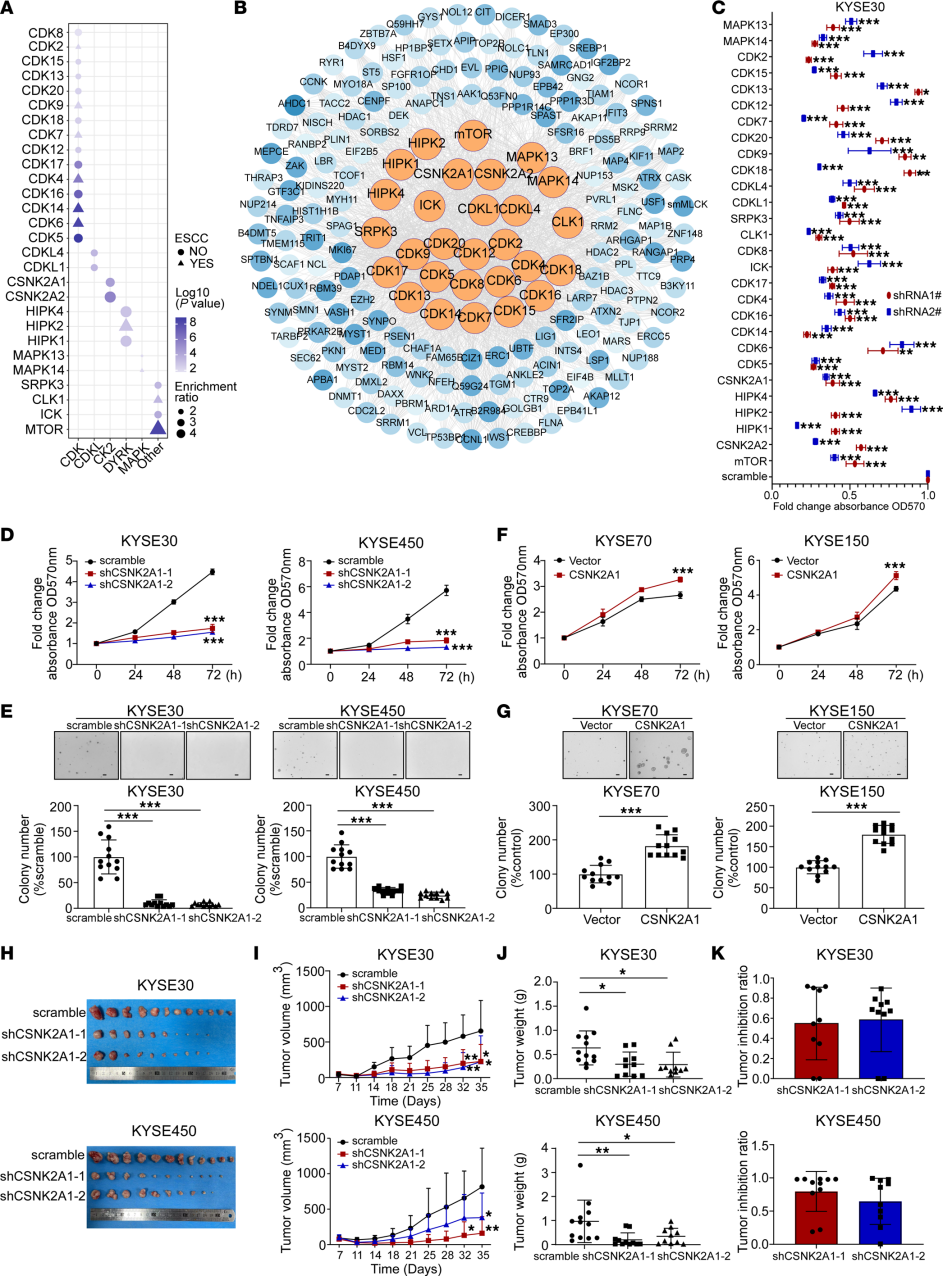
Identification of kinases and their potency in ESCC. (**A**) Potential activated kinases predicted by a kinase-substrate phosphorylation network are depicted. Dots represent previously unstudied kinases in ESCC, and triangles represent kinases implicated in ESCC based on previous literature. The color denotes the calculated *P* value for each kinase, and the size of the shapes corresponds to their enrichment ratios. (**B**) Kinase-phosphosubstrate regulation networks are depicted for selected kinases and their substrates, with orange circles representing kinases and blue circles representing their corresponding substrates. (**C**) Cell proliferation at 72 hours was evaluated by MTT assay after knockdown of the indicated kinases in KYSE30 cell (*n* = 6 for each group). (**D** and **E**) Cell proliferation measured by MTT assay (*n* = 6 for each group) (**D**) and soft agar assay (*n* = 12 for each group) (**E**) after CSNK2A1 knockdown. Scale bar: 200 μm. (**F** and **G**) Cell proliferation measured by MTT (*n* = 6 for each group) (**F**) and soft agar (*n* = 12 for each group) (**G**) assays after CSNK2A1 overexpression. Scale bar: 200 μm. (**H**–**K**) KYSE30 and KYSE450 cells stably expressing scramble or shCSNK2A1 were s.c. injected into the right flank of each mouse (KYSE30: scramble, *n* = 12; sh1, *n* = 10; sh2, *n* = 10; KYSE450: scramble, *n* = 12; sh1, *n* = 11; sh2, *n* = 10). Tumors were excised at the end of the experiment. Images of xenograft tumors are shown in **H**, while **I** presents xenograft tumor growth curves data from mice experiments; **J** displays tumor weights; and **K** represents tumor inhibition ratios. In all statistical plots, data were expressed as the mean ± SD. Two-tailed Student’s *t* test (**F** and **G**) and 1-way ANOVA (**C**–**E**, **I**, and **J**) were used to determine statistical significance. *P < 0.05, **P < 0.01, ***P < 0.001. Representative results from at least 3 independent biological replicates (**C**–**G**) are shown.

To predict candidate kinases associated with altered phosphorylation networks in ESCC, we performed kinase activity analysis using the in silico kinome activity profiling (iKAP) algorithm (45). Our analysis suggested that a total of 28 activated kinases were closely linked to the altered phosphorylation networks (Figure 5, A and B, and Supplemental Table 6). Among these activated kinases, mTOR, CLK1, and CDK7 have been previously reported (21, 46, 47) in ESCC. To investigate the roles of these kinases in ESCC cell proliferation, we generated KYSE30 and KYSE450 knockdown cells with shRNAs that targeted candidate kinases separately. Our results reveal that depletion of the majority of kinase transcripts significantly impaired cell proliferation (Figure 5C and Supplemental Figure 5D). Notably, knockdown of CSNK2A1 resulted in the most pronounced inhibition of cell proliferation in both cell lines. CSNK2A1, a catalytic subunit of CK2, has been identified as a potential therapeutic target in acute myeloid leukemia (48, 49). Therefore, we conducted MTT, soft agar, and colony formation assays to determine the effect of CSNK2A1 knockdown or overexpression on ESCC cell proliferation. The efficiency of CSNK2A1 knockdown and overexpression was confirmed by Western blotting (Supplemental Figure 5, E and G). Our findings reveal that knockdown of CSNK2A1 dramatically reduced the growth of KYSE30 and KYSE450 cells (Figure 5, D and E, and Supplemental Figure 5F), while overexpression of CSNK2A1 promoted the growth of KYSE70 and KYSE150 cells (Figure 5, F and G, and Supplemental Figure 5H). To investigate whether CSNK2A1 contributes to ESCC tumor growth in vivo, a CDX model was also established. Our results demonstrate that depletion of CSNK2A1 profoundly impeded tumor growth in vivo (Figure 5, H–K). Collectively, these findings highlight the potential of CSNK2A1 as a therapeutic target for ESCC.

Histone deacetylase 1 (HDAC1) is a nuclear enzyme involved in transcriptional repression, and the phosphorylation of HDAC1 at Ser421 and Ser423 by CK2 plays a crucial role in regulating enzymatic activity, complex formation, and transcriptional repression (50). To validate our kinase prediction results, we performed Western blotting on ESCC tissue samples to assess the levels of p-HDAC1 S421/S423. Consistent with our sequencing data, we found elevated level of p-HDAC1 S421/S423 in tumor tissues (Supplemental Figure 6A). These results suggest that CSNK2A1 may initiate downstream substrate phosphorylation events, as visualized within the kinase-substrate network (Figure 5B). We also observed that knockdown of HDAC1 inhibited the cell proliferation of KYSE30 and KYSE450 cells (Supplemental Figure 6, B–D). To investigate the function of the p-HDAC1 S421/S423 sites, we generated phosphorylation-deficient double mutants (denoted as HDAC1 S421/S423A) and phosphorylation-mimetic double mutants (denoted as HDAC1 S421/S423D) in KYSE70 and KYSE150 cells. MTT and colony formation assays revealed that HDAC1 S421/S423A mutant cells exhibited lower proliferation and colony formation abilities than HDAC1-WT and HDAC1 S421/S423D mutant cells (Supplemental Figure 6, E and F). Subsequently, we investigated the effect of HDAC1 S421/S423 phosphorylation on HDAC1 enzymatic activity. Our results reveal that HDAC1-S421/S423A mutant cells exhibited decreased HDAC1 enzymatic activity compared with that of HDAC1-WT and HDAC1-S421/S423D mutant cells (Supplemental Figure 6G). It has been reported (48) that EGFR-mediated activation of ERK2 can facilitate the phosphorylation of CK2 at T360/S362 and can, thus, enhance CK2 activity. To explore the function of the CSNK2A1 T360/S362 sites in regulating HDAC1 activity, we generated CSNK2A1 S421/S423A and CSNK2A1 S421/S423D mutant cells. In vitro kinase assay results indicate that the CSNK2A1 S421/S423 sites play a crucial role in activating HDAC1 (Supplemental Figure 6H). Furthermore, we found that, compared with CSNK2A1-WT and CSNK2A1 S421/S423D mutant, CSNK2A1 S421/S423A suppressed cell proliferation and colony formation abilities (Supplemental Figure 6, I and J).

To further elucidate the molecular function of CSNK2A1 in ESCC, we conducted a comparative analysis of protein expression between the high- and low-CSNK2A1–expression groups (Supplemental Figure 7A). Furthermore, enrichment analysis revealed that crucial pathways involved in cell proliferation, including DNA replication and cell cycle, were enriched in the group with high CSNK2A1 expression (Supplemental Figure 7B and Supplemental Table 7). In the CSNK2A1 high-expression group, we noted several highly expressed proteins that have been previously implicated (34, 51–53) in ESCC development, including IGFBP3 (Wilcoxon test, *P* = 0.014), LAMC2 (Wilcoxon test, *P* = 0.0042), MAGEA4 (Wilcoxon test, *P* = 0.05), and SLC7A5 (Wilcoxon test, *P* = 7.9 × 10^–4^) (Supplemental Figure 7C). Additionally, we found some proteins in the CSNK2A1 high-expression group were correlated with patient survival, including CPOX (Wilcoxon test, *P* = 2.2 × 10^–6^; Log-rank test, *P* = 0.0017), DSC3 (Wilcoxon test, *P* = 2.6 × 10^–4^; Log-rank test, *P* = 0.071), PKP1 (Wilcoxon test, *P* = 6.9 × 10^–6^; Log-rank test, *P* = 0.042), and RPLP1 (Wilcoxon test, *P* = 0.0016; Log-rank test, *P* = 0.047) (Supplemental Figure 7D).

### Inhibitory effect of CSNK2A1 in vitro and in vivo.

**Figure 6:**
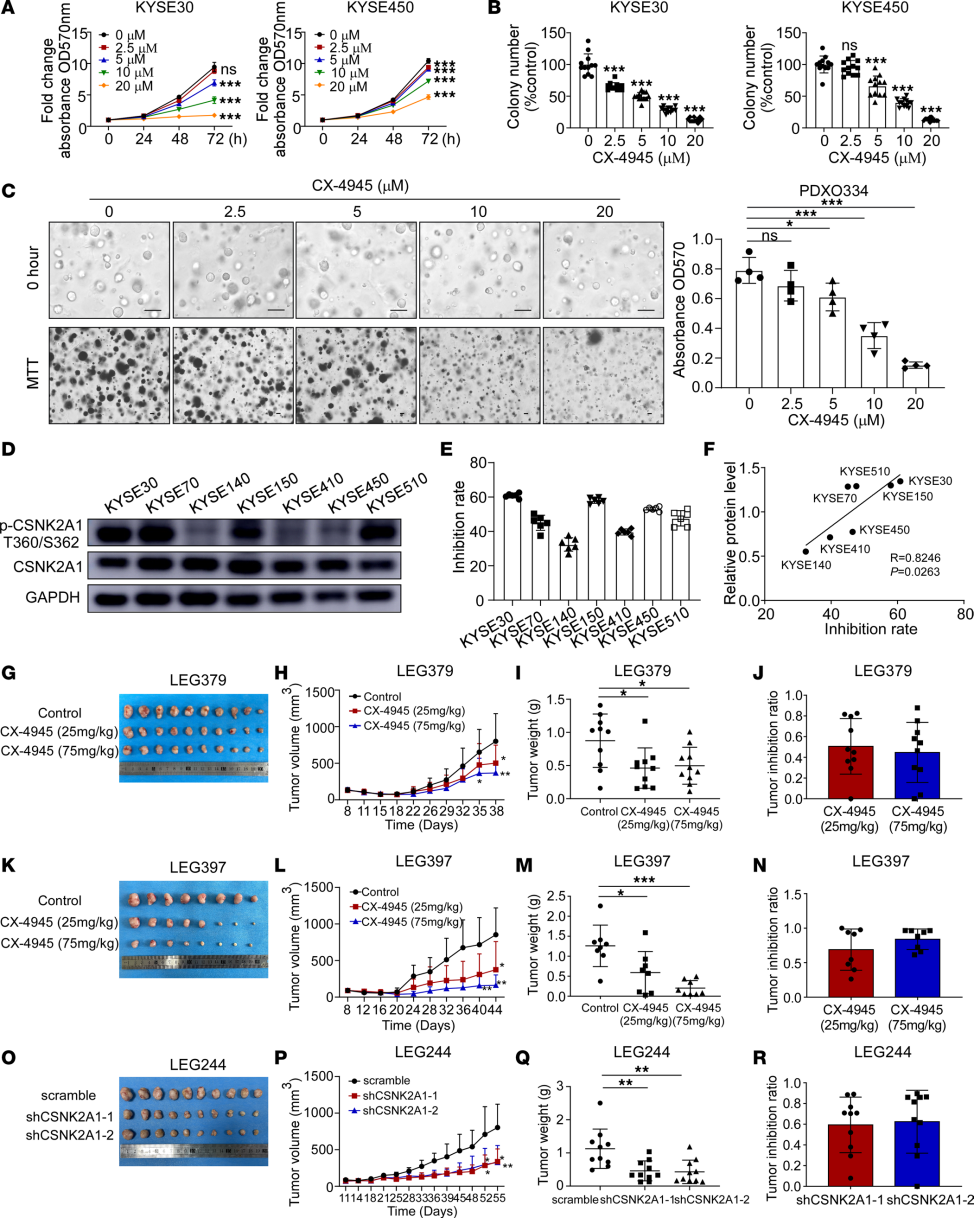
Tumor inhibitory effect of CX-4945 in vitro and in vivo. (**A** and **B**) MTT (*n* = 6 for each group) (**A**) and soft agar (*n* = 12 for each group) (**B**) assays were performed on KYSE30 and KYSE450 cells following treatment with CX-4945 at concentrations ranging from 0 μM to 20 μM. (**C**) The growth of organoids was evaluated after treatment with CX-4945 at the same concentration (*n* = 4 for each group). Scale bar: 100 μm. (**D**) Western blotting was performed to determine the protein levels of CSNK2A1 and p-CSNK2A1 T360/S362 in ESCC cell lines. (**E**) The inhibitory effect of CX-4945 on cell proliferation was assessed using MTT assay in ESCC cell lines. (**F**) Pearson correlation analysis was employed to evaluate the correlation between p-CSNK2A1 T360/S362 levels and inhibition rate of cell proliferation in 7 ESCC cell lines. (**G**) LEG379 tumors excised from the mice treated with CX-4945 were analyzed (*n* = 10 for each group). (**H**–**J**) PDX tumor growth (**H**), tumor weights (**I**), and tumor inhibition ratios (**J**) of the LEG379 case treated with CX-4945 were determined. (**K**–**N**) LEG397 tumors excised from mice treated with CX-4945 were examined (*n* = 8 for each group) (**K**), followed by analysis of PDX tumor growth (**L**), tumor weights (**M**), and tumor inhibition ratios (**N**) of the LEG397 case. Additionally, LEG244 tumors obtained from mice treated with lentiviruses encoding shCSNK2A1 were investigated (*n* = 10 for each group). (**O**–**R**) The LEG244 case treated with lentiviruses encoding shCSNK2A1 were examined (**O**), and PDX tumor growth (**P**), tumor weights (**Q**), and tumor inhibition ratios (**R**) were determined. In all statistical plots, data were expressed as the mean ± SD. One-way ANOVA (**A**–**C**, **H**, **I**, **L**, **M**, **P**, and **Q**) was used to determine statistical significance. *P < 0.05, **P < 0.01, ***P < 0.001. Representative results from at least 3 independent biological replicates (**A**–**E**) are shown.

Silmitasertib (CX-4945) is a potent and orally bioavailable CK2 inhibitor with broad-spectrum anticancer activity (54). Clinical trials have been conducted to evaluate the efficacy of CX-4945 in the treatment of cholangiocarcinoma (NCT02128282), COVID-19 (NCT04663737), coronavirus (NCT04668209), and other diseases. To investigate the potential therapeutic effects of targeting CSNK2A1 with CX-4945 on ESCC cell growth, we performed MTT, soft agar, and organoid culture assays. CX-4945 effectively inhibited cell proliferation and organoid growth in a dose-dependent manner (Figure 6, A–C, and Supplemental Figure 8, A–C). Subsequently, we examined whether the effect of CX-4945 on ESCC growth was dependent on the levels of CSNK2A1 or p-CSNK2A1 T360/S362. We analyzed the correlation between CSNK2A1 or p-CSNK2A1 T360/S362 levels and the inhibition rates of cell proliferation (Figure 6, D and E). The results demonstrate that ESCC cells with high p-CSNK2A1 T360/S362 levels were more sensitive to CX-4945 treatment (Figure 6F). Furthermore, we evaluated the therapeutic efficacy of CX-4945 using ESCC patient-derived xenograft (PDX) mouse models derived from LEG379, LEG397, and LEG460 cases with relatively elevated or moderate p-CSNK2A1 T360/S362 levels (Supplemental Figure 8, D and E). Our results demonstrate that the tumor volumes (Figure 6, G and H) and weights (Figure 6, I and J) of the LEG379 tumors decreased upon CX-4945 treatment. Similarly, CX-4945 treatment also inhibited LEG397 and LEG460 tumor growth (Figure 6, K–N, and Supplemental Figure 8, F–I).

To elucidate the specific contribution of CSNK2A1 to tumor growth, we conducted an in vivo knockdown experiment using LEG244 PDX model mice by employing lentivirus-mediated shRNA delivery targeting CSNK2A1 (Supplemental Figure 8E). Our results demonstrate a reduction in tumor volumes and weights upon knockdown of CSNK2A1 compared with those in the scramble group, resulting in an approximately 60% suppression rate (Figure 6, O–R). To further validate the effect of p-CSNK2A1 T360/S362 on tumor growth in ESCC, we measured expression and phosphorylation levels of CSNK2A1 and HDAC1 in tumor samples obtained from our PDX models by Western blotting. Our results indicate that CX-4945 treatment or CSNK2A1 knockdown led to a reduction of p-CSNK2A1 T360/S362 and p-HDAC1 S421/S423 levels in these PDX models (Supplemental Figure 8J). In summary, these findings underscore the potential importance of targeting CSNK2A1 for ESCC treatment.

## Discussion

In this study, we conducted a comprehensive omics analysis that revealed the molecular alterations in ESCC and identified potential prognostic molecules and therapeutic targets. Despite substantial advancements achieved through large-scale omics profiling of ESCC, previous phosphoproteomics analyses were limited by sample scarcity (20, 21, 55). We overcame this limitation in our research by utilizing a larger number of paired patient tissues to generate transcriptomics, proteomics, and phosphoproteomics data sets. In our study, correlations between transcriptomics and proteomics data were investigated, revealing alterations in various key metabolic pathways. Clustering analysis of the proteomics data revealed 3 distinct molecular subtypes of ESCC. Subsequent analysis of these subtypes revealed TIMMDC1 as a potential prognostic molecule for ESCC. Additionally, the phosphoproteomics analysis highlighted CSNK2A1 as a potential therapeutic target for ESCC.

Determining the correlations between mRNA and protein products has facilitated the investigation of downstream signaling consequences resulting from genetic alterations in various cancers (14, 15, 18, 56–61). Our analysis revealed more robust gene-wise and sample-wise correlations in tumor tissues than in nontumor tissues. Moreover, our observations indicate that the key pathways altered in ESCC are predominantly involved in metabolic processes. Additionally, our analysis revealed that the differentially expressed proteins showed enrichment in pathways related to DNA replication, spliceosome, and immune-related signaling pathways. These findings are consistent with previous studies (20, 62) on esophageal and gastric cancer. Moreover, the functions of some differentially expressed proteins identified in our study have been previously investigated (46, 63–68); several EMT-related proteins, such as WNT5A, MMP1, MMP2, MMP14, LAMC2, FN1, and COL5A2, have been found to enhance cancer cell migration and invasion, suggesting their potential involvement in metastasis. Furthermore, certain cell cycle–related proteins, including TOP2A, PCNA, MCM2, MCM4, MCM5, MCM6, and MCM7, have also been reported (69–71) to facilitate cancer cell proliferation, and the enhanced expression of these cell cycle–related proteins may contribute to uncontrolled ESCC cell proliferation. Collectively, these transcriptomics and proteomics analyses provide valuable insights into the molecular alterations underlying ESCC progression.

Molecular subtyping of cancers can aid patient stratification based on precise molecular signatures to achieve personalized diagnosis and treatment planning. Currently, liquid chromatography–tandem mass spectrometry (LC-MS/MS) proteomics data have been utilized in several studies (57, 60, 62, 72–77) to perform molecular subtyping of various cancer types, including gastric cancer, lung cancer, early-stage hepatocellular carcinoma, head and neck squamous cell carcinoma, pancreatic ductal adenocarcinoma, colorectal cancer, and breast cancer. Additionally, molecular subtyping of ESCC patient-derived tissue samples has been performed based on gene mutation and transcription profiling data (78, 79). Our proteomics analysis classified 60 patient tissue samples into 3 distinct subtypes. Among these subtypes, patients with S-III exhibited the poorest survival outcome. Notably, a study (21) conducted by Li et al. also classified patients into 3 subtypes; however, their follow-up period (approximately 2 years) was shorter than that of our study (7 years). Therefore, we considered that our protein-based subtype classification using a longer follow-up period more accurately reflects the clinical status of patients and provides better guidance for treatment decisions. Consistent with previous investigations (20, 21), we found that the cell cycle, E2F targets, EMT, and spliceosome-related pathways were important for maintaining the ESCC phenotype. Furthermore, our analysis revealed that S-I exhibited enrichment of proteins involved in fatty acid metabolism, adipogenesis, and reactive oxygen species. S-II showed enrichment of proteins associated with Hedgehog signaling, apical junction, and coagulation. Moreover, S-III was characterized by an enrichment of proteins involved in glycolysis and DNA repair. Additionally, we discovered several actionable protein targets — such as SPARC, COL1A1, ADH1B, L1CAM, S100A7, PCNA, and EGFR — within 3 subtypes. These targets can be effectively modulated using FDA-approved drugs. Furthermore, ongoing investigations are exploring the potential of MMP14, CASP7, and PRMT1 as candidate drug targets (80). Collectively, these findings provide complementary information regarding the molecular subtypes of ESCC and their potential clinical implications for future therapeutic interventions.

A previous study (81) reported that TIMMDC1 interacts with multiple members of the mitochondrial complex I assembly factor family and core complex I subunits. Inhibition of TIMMDC1 in HeLa cells resulted in decreased cellular respiration, complex I activity, and complex I subunit stability (40). Additionally, depletion of TIMMDC1 inhibited migration and proliferation in 95D lung carcinoma cells, leading to a marked reduction in mitochondrial viability, membrane potential, and ATPase activity within these cells (82). Moreover, researchers have observed that tumor growth is inhibited due to impaired efficiency of the electron transport chain and oxidative mitochondrial metabolism facilitated by complex I (83). Another study (84) suggested that metformin can suppress tumor growth by inhibiting cellular respiration and mitochondrial complex activity. Our study revealed a significant association between elevated levels of TIMMDC1 and poor prognosis. Moreover, knockdown of TIMMDC1 led to inhibition of cell growth both in vitro and in vivo. These experimental findings validate the prognostic value of TIMMDC1 and suggest its potential as a therapeutic target for ESCC.

A key finding of this study is the identification of pivotal kinases with therapeutic potential for ESCC. We selected 28 candidate kinases for experimental perturbation to determine their contributions to ESCC cell proliferation. Among the identified kinase candidates, mTOR, CLK1, and CDK7 have been previously implicated (21, 46, 47) in ESCC development. Our study reveals that inhibition of CSNK2A1 attenuated cell proliferation and suppressed ESCC tumor growth in vivo. Previous studies (85–89) have demonstrated that CSNK2A1 promotes tumorigenesis by enhancing several oncogenic signaling pathways in various malignancies, including breast cancer, lung cancer, kidney cancer, colorectal cancer, and prostate cancer. Additionally, CSNK2A1 promotes the invasion and migration of cells by regulating the EMT, NF-κB, and PI3K/Akt/mTOR signaling pathways (90). Our data analysis revealed proteins associated with elevated CSNK2A1 levels in ESCC that were also correlated with tumor maintenance in other cancer types. For instance, CPOX autoantibodies were identified as discriminatory markers between prostate cancer and healthy tissues (91). DSC3 was identified as a highly specific marker for squamous cell carcinoma (92). Moreover, DSC3 was downregulated in colorectal cancer cells, and its methylation status was shown to be an effective prognostic marker (93). Furthermore, CX-4945 is the first CK2-specific inhibitor under clinical evaluation for its efficacy against human solid and hematological tumors (54). Our findings indicate that CX-4945 exhibits inhibitory effects on ESCC both in vivo and in vitro, suggesting the potential of CSNK2A1 as a promising therapeutic target.

There are several limitations in our study. Firstly, our cohort consisted of only 60 paired clinical samples; a larger clinical cohort would be more conducive to illustrating the molecular characteristics of ESCC. Secondly, although we predicted several proteins as potential prognostic molecules, we validated only 1 protein (TIMMDC1) from the candidate list. Other proteins may also serve as effective prognostic molecules of ESCC. Regarding TIMMDC1, we have only validated its prognostic potential in a limited number of tissue samples; therefore, more studies using larger sample sizes are necessary to explore its clinical relevance and elucidate the underlying molecular mechanisms. Finally, although our method successfully predicted several kinases, among which CSNK2A1 exhibited a superior inhibitory effect on tumor growth, it is worth considering the utilization of CK2-related drugs for functional verification in future studies on ESCC using additional PDX models, given their ongoing clinical investigation.

In summary, the multi-omics integrative analysis represents a valuable and potent tool that offers a complementary and comprehensive understanding of ESCC, thereby providing an opportunity to facilitate the translation of fundamental research into more precise diagnostic methods and effective treatments.

## Methods

### Sex as a biological variable.

For studies involving humans and animal models, sex was not considered as a biological variable.

### Patient samples.

The clinical samples used in this multi-omics study were collected from patients at Linzhou Cancer Hospital (Anyang, China) between December 2013 and September 2014. Tumor specimens along with their corresponding adjacent nontumor tissue samples (defined as those located more than 5 cm away from the tumor margin) were obtained. No preoperative therapy was administered to any of the patients in our cohort. Fresh tissues obtained from surgery were divided into 3 sections: the first section was fixed with formalin for H&E staining; the second section was treated with RNA stabilization solution, stored at –80°C, and utilized for RNA extraction; and the third section was immediately frozen in liquid nitrogen for 1 hour and subsequently stored at –80°C until protein extraction. Each sample was assigned a unique identification code according to its original record number. Formalin-fixed and paraffin-embedded specimens were generated for each tissue, while the H&E-stained slides were independently evaluated by 2 experienced pathologists who provided information regarding tumor purity, TNM stage, pathological type, and lymphatic status. Patient details, including surgery date, age, sex, smoking history, drinking history, and family history, were recorded. Annual follow-up examinations were performed over a period of 7 years.

The TIMMDC1 and p-HDAC1 S421/S423 protein levels were measured in additional ESCC tissues and their corresponding adjacent nontumor tissues, which were freshly collected at Linzhou Cancer Hospital using the same methodology as described above.

### Cell lines.

The human ESCC cell lines KYSE30, KYSE70, KYSE140, KYSE150, KYSE410, KYSE450, and KYSE510 were obtained from the Type Culture Collection of the Chinese Academy of Sciences. The human immortalized normal esophageal epithelial cell line (SHEE) was donated by Enmin Li from the Laboratory of Tumor Pathology (Shantou University Medical College, Shantou, China). 293T cells were acquired from the American Type Culture Collection (ATCC). All cell lines used in this study were cytogenetically tested. Human ESCC cells were cultured in RPMI-1640 medium supplemented with penicillin (100 units/mL), streptomycin (100 μg/mL), and 10% FBS (Biological Industries). 293T cells were maintained in DMEM supplemented with 10% FBS. All cells were kept at 37°C in an incubator containing 5% CO_2_.

### Reagents and antibodies.

CX-4945 was purchased from Lollane Biotechnology. The antibodies used in this study included anti-TIMMDC1 (ABclonal, A15839); anti-CSNK2A1 (Proteintech, 10992-1-AP); anti-HDAC1 (Proteintech, 10197-1-AP); anti–p-CSNK2A1 T360/S362 (ABclonal, AP0335); anti–p-HDAC1 S421/S423 (Thermo, PA5-36911); and anti-GAPDH (Proteintech, HRP-60004).

Details for RNA-Seq and LC-MS/MS are described in the Supplemental Methods.

### Pathway enrichment analysis.

The 2-sided hypergeometric test was adopted for the KEGG pathway enrichment analysis of differentially expressed proteins or phosphoproteins. For each type, we defined the following: *N*, number of human proteins annotated by at least 1 term; *n*, number of human proteins annotated by term t; *M*, number of upregulated or downregulated proteins annotated by at least 1 term; and *m*, number of upregulated or downregulated proteins annotated by term t.

The enrichment ratio was then computed, and the *P* value was calculated based on the hypergeometric distribution as follows:


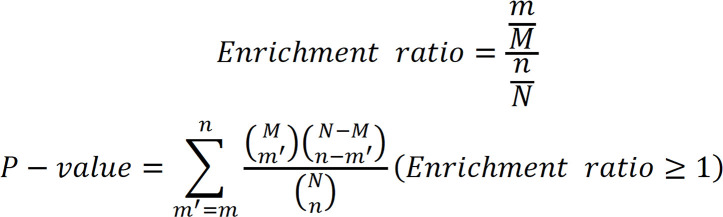
 (Equation 1)

or


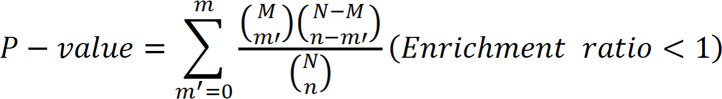
 (Equation 2)

Furthermore, we performed single-sample gene set variation analysis (ssGSEA) using 50 cancer hallmark gene sets collected from the MSigDB to identify the pathway alterations in each subtype. The protein expression matrix was utilized to calculate the ssGSEA scores for each gene set. Normalized enrichment scores (NES) and *P* values were calculated using 1,000 permutations. At least 1 subtype with an adjusted *P* < 0.01 was regarded as significantly enriched, and the average NES value was then calculated as the enrichment score (ES) of the proteomic subtypes (94).

### Consensus clustering analyses.

Prior to clustering analysis, differentially expressed proteins were identified. To identify proteomic subtypes of ESCC, consensus clustering (R package ConsensusClusterPlus version. 1.54.0) — a reconciling clustering algorithm with a resampling strategy — was implemented. The basic parameters were set as follows: maximum up to 6 subtypes (maxK = 6), 5,000-time repetitions (reps = 5,000), and resampling 95% of the samples (pItem = 0.95). Three clustering algorithms with corresponding distance functions were used to obtain the potential subtypes that were significantly associated with overall survival (OS) outcomes. In the k-means (KM) clustering, Euclidean distance was applied; in the hierarchical clustering (HC) and partitioning around the medoid (PAM), 7 distance functions comprising Pearson (1 – Pearson correlation), Spearman (1 – Spearman correlation), Euclidean, binary, maximum, Canberra, and Minkowski were applied. Finally, the optimal subtype for proteins and the corresponding signature proteins were determined based on the PAM with Canberra.

### Phosphoproteomics data analysis.

For the phosphoproteomics data, all the intensities of the phosphopeptides of 60 patient samples were exported from MaxQuant software (v1.6.1.0). The confidently identified phosphopeptides (with a 1% FDR and 75% localization probability) were normalized using the same strategy used for proteome analysis with the corresponding proteomics data sets. The iKAP algorithm was applied to estimate kinase activity using the ratios of identified phosphosites with cutoff ratios of 1.45 between tumor and nontumor tissues. iKAP estimates change in a kinase’s activity by calculating and summing all the identified substrates instead of only a single substrate, which could mitigate the bias from inherent noise of phosphoproteomics data (45). The estimation of kinase activities is based on the consensus phosphorylation motif of a kinase, while iGPS integrates publicly available databases such as PhosphoSite and Phospho.ELM to store information on all kinase-substrate networks and substrate motifs (95).

### Correlations between mRNA and protein expression.

To determine the correlations between the transcriptomics and proteomics data of patients with ESCC, Spearman’s correlation coefficients of genes/proteins were calculated using both RNA-Seq and MS data (5,676 genes) obtained from the tumor or paired nontumor tissues. Additionally, gene set enrichment analysis (GSEA) was performed to identify pathway alterations. The GSEA scores were calculated based on the correlation scores obtained from tumor and nontumor tissues (56).

### Survival curves.

We utilized Kaplan-Meier analysis to estimate the OS of patients with different proteomic subtypes. The Cox proportional hazard model was used to evaluate the effect of protein expression on survival time. Prior to conducting the Log-rank test for a given protein, patients were classified into high and low groups based on their quantile-normalized protein levels in tumor tissues (< first quartile and > third quartile, respectively). Subsequently, the corresponding Kaplan-Meier survival curves for OS were generated based on the cutoff point.

### Potential prognostic biomarkers.

The following 4 criteria were used to identify potential biomarkers: (a) candidate proteins were identified in all 60 tumor samples; (b) these candidate proteins exhibited high abundances, ranking among the top 1,000 proteins listed and displaying differential expression in tumor and adjacent nontumor tissues; (c) the candidate proteins were highly expressed in S-III; and (d) highly expressed candidates were negatively correlated with OS (HR > 1.5 and Kaplan-Meier analysis, Log-rank test, *P* < 0.05).

### IHC.

The human ESCC TMA (HEsoS180Su08) was obtained from Shanghai Outdo Biotech Company. Formalin-fixed, paraffin-embedded tumor and adjacent nontumor samples were sectioned, deparaffinized, and hydrated. The TMA was subjected to antigen retrieval by boiling in sodium citrate buffer (10 mM/L, pH 6.0) for 90 seconds. Subsequently, the TMA was treated with 3% H_2_O_2_ for 5 minutes. Blocking was then performed using goat serum for 60 minutes before incubating overnight at 4°C with the specific primary antibody. The following day, the TMA was incubated with the secondary antibody for 30 minutes at room temperature (RT). Streptavidin working solution labeled with horseradish enzyme was applied to the TMA, which was then stained with DAB solution (ZSGB-BIO, ZLI-9018); the reaction was terminated upon observation of color development under a microscope. Finally, counterstaining was conducted, and the data were analyzed by using Aperio Image Scope software (v12.3.2.8013).

### Western blot.

The cells or tissues were collected and treated with RIPA lysis buffer (Solarbio, R0020) supplemented with protease and phosphatase inhibitors on ice. The lysate was gently pipetted to ensure complete cell lysis. Subsequently, the cell suspension was centrifuged at 14,000*g* for 15 minutes at 4°C, and the resulting supernatant was transferred to a fresh tube. The protein concentration was determined using a BCA kit. The proteins were separated by SDS-PAGE and subsequently transferred onto PVDF membranes. Following the transfer, the membranes were blocked with 5% skim milk for 1 hour at RT. Next, the membrane was incubated with a specific primary antibody overnight at 4°C. The following day, the membranes were washed 3 times in TBST and then incubated with an HRP-conjugated secondary antibody for 2 hours at RT. Finally, after the membranes were washed 3 times with TBST, ECL reagent (Meilunbio, MA0186-1) was added to visualize the specific bands.

### shRNA.

For gene knockdown, lentiviruses were generated by transfecting 293T cells with the shRNA plasmid, pMD2.G, and pSPAX2. The shRNA sequences cloned into the plasmids are provided in Supplemental Table 8. After transfection for 24 hours and 48 hours, the virus-enriched medium was collected, filtered through a 0.45 μm filter, and stored at 4°C for up to 1 week. Subsequently, ESCC cell lines were treated with lentivirus-enriched medium supplemented with polybrene (Sigma-Aldrich, TR-1003-G) for 24 hours. Afterward, the media were replaced with RPMI 1640 containing puromycin (Solarbio, P8230), and the cells were incubated for 1–2 days to select positive cells.

### Overexpression.

For gene overexpression, we constructed the overexpression plasmids PCDH-3×Flag-TIMMDC1 and PCDH-3×Flag-CSNK2A1. Transient transfection of the aforementioned plasmids was subsequently performed in the KYSE70 and KYSE150 cell lines. After 48 hours of transfection, the cells were harvested for Western blot analysis to evaluate the effect of overexpression.

### Organoid culture.

Esophageal PDX-derived organoids (PDXOs), derived from LEG334 and LEG352 ESCC PDX model tumors, were established using previously published protocols (96). The minced tumor tissue was incubated with buffer containing Y-27632 (MCE, HY-10071) and collagenase IV (Thermo Fisher Scientific, 17104019). Subsequently, the tissue fragments were filtered, and the collected cell suspension was centrifuged at 4,000*g* for 5 minutes at 4°C and resuspended in Matrigel (Corning, 356231) before being dispensed into the wells of a 24-well plate. Once the resuspended cells solidified, they were cultured in organoid medium (Thermo Fisher Scientific, 1234028) for 10–14 days. The cells were passaged at least 3 times for organoid formation.

For PDXO treatment, the organoid cells were transferred to a 96-well plate and cultured for 7 days. Images were acquired prior to and after CX-4945 treatment at concentrations ranging from 0 μM to 20 μM. After incubation for 96 hours, an MTT assay was performed, and the OD values at 570 nm were obtained to assess the efficacy of CX-4945.

### HDAC1 activity assay.

The WT HDAC1 plasmid (PCDH-3×Flag-HDAC1) or mutant HDAC1 plasmids (PCDH-3×Flag-HDAC1 S421/S423D and PCDH-3×Flag-HDAC1 S421/S423A) were transfected into KYSE70 or KYSE150 cells, respectively. Subsequently, the WT and mutant HDAC1 cells were harvested for quantification of HDAC activity using an HDAC1 colorimetric activity assay kit (GENMED, GMS50082.2). The calculation of HDAC1 activity was performed according to the manufacturer’s recommendation.

### In vitro kinase assay.

For in vitro kinase assays, WT CSNK2A1 plasmid (PCDH-3×Flag-CSNK2A1)or mutant CSNK2A1 plasmids (PCDH-3×Flag-CSNK2A1 T360/S362D and PCDH-3×Flag-CSNK2A1 T360/S362A) were transfected into 293T cells. Subsequently, WT and mutant CSNK2A1 proteins were purified from 293T cells after 48 hours. Recombinant HDAC1 proteins were purified from *E*. *coli*. Subsequently, WT CSNK2A1 (100 ng) or mutant CSNK2A1 (100 ng) was incubated with 1 μg of recombinant WT HDAC1 protein and 250 μM ATP, respectively. The reactions were carried out in kinase buffer for 30 minutes at 30°C and were stopped by using a 6 × SDS sample buffer. Proteins were separated by SDS-PAGE and analyzed by Western blotting.

The methods used for the MTT, colony formation, and soft agar assays are described in the Supplemental Methods.

### CDX.

The CDX murine models were generated using the KYSE30 and KYSE450 cell lines. First, 6-week-old female Nu/Nu mice (SPF Biotechnology Co.) were randomly divided into 3 groups: a scramble group, an sh1 group (shTIMMDC1 or shCSNK2A1 group), and an sh2 group (shTIMMDC1 or shCSNK2A1 group). For the KYSE30 cells, each group was s.c. injected with 5 × 10^6^ stably transfected scramble cells, sh1 cells, or sh2 cells. Similarly, for the KYSE450 cells, 3 groups were s.c. injected with 1 × 10^7^ stably transfected scramble cells, sh1 cells, or sh2 cells. Tumor volumes were measured twice weekly and calculated using the following formula: V = (length) × (width) × (width)/2. Mice were euthanized when the tumor size reached approximately 1,000 mm^3^. Tumor growth curves and tumor inhibition rates were calculated based on the recorded data.

### PDX.

The LEG379, LEG397, and LEG460 cases were utilized to evaluate the effects of CX-4945 treatment. Approximately 0.08 g of tumor tissue was implanted into 6-week-old female NOD-SCID mice (SPF Biotechnology Co.) for each case. After 1 week, the NOD-SCID mice were separated into the control group, the 25 mg/kg group, and the 75 mg/kg group based on tumor volumes and body weights. CX-4945 was orally administered twice daily. Tumor volumes were assessed twice weekly utilizing the following formula: V = (length) × (width) × (width)/2. Mice were euthanized when the tumor size reached approximately 1,000 mm^3^. The tumor growth curves and tumor inhibition rates were calculated based on the recorded data.

The LEG244 case was utilized to assess the effect of lentivirus-mediated gene silencing. After the implantation of tumor fragments for 1 week, the mice were divided into 3 groups: the scramble group, the shCSNK2A1-1 group, and the shCSNK2A1-2 group. The lentivirus-enriched media were subjected to ultracentrifugation at 56,000*g* for 3 hours at 4°C. Intratumoral injections of scramble virus, shCSNK2A1-1 virus, or shCSNK2A1-2 virus were administered twice weekly over a period of 6 weeks. Tumor growth curves and inhibition rates were calculated as indicated above.

### Statistics.

The following statistical analysis methods were utilized for single-omic and multi-omics data, experimental data, and clinical data. Standard 2-sided statistical tests, including the Log-rank test, 2-tailed Student’s *t* test, Fisher’s exact test, Spearman’s correlation, Wilcoxon test, Hypergeometric test, and 1-way ANOVA, were used. To account for multiple-testing, adjusted *P* values were calculated using the Benjamini-Hochberg (BH) correction method. For experimental validation, each assay was repeated independently at least 3 times, and the results are presented as mean ± SD. Statistical significance was determined by 2-tailed Student’s *t* test and 1-way ANOVA. *P* < 0.05 was considered significant.

### Study approval.

The use of clinical samples was received the approval of the Ethics Committee of China-US (Henan) Hormel Cancer Institute (no. CUHCIH2015001), and informed consent was obtained from all participants who volunteered for the study. The animal experiments conducted in this study were approved by the Ethics Committee of China-US (Henan) Hormel Cancer Institute (no. CUHCI2021002).

### Data availability.

All original proteomics and phosphoproteomics data of this study were deposited to iProX (ProteomeXchange ID: PXD035562). All original of RNA-Seq data of this study were deposited to SRA of NCBI (PRJNA861875). Analytic code for this study can be made available by the corresponding author. Values for all other data are available in the Supporting Data Values file.

## Author contributions

ZD and KL conceived and designed the study; DZ and YG developed methodology; YG and DZ performed software; YG, DZ, and WN performed formal analysis; GH, ZL, and JW provided resources; DZ, XJ, YZ, LH, and PW performed validation; DZ, HW, MHL, and KVL wrote original drafts; DZ and YG provided visualization; ZD and KL supervised the study; ZD, KL, and YG provided funding. The order of co–first authors was based on relevance of contributions to this project.

## Supplementary Material

Supplemental data

Unedited blot and gel images

Supplemental table 1

Supplemental table 2

Supplemental table 3

Supplemental table 4

Supplemental table 5

Supplemental table 6

Supplemental table 7

Supplemental table 8

Supporting data values

## Version 1 04/23/2024

In-Press Preview

## Version 2 05/22/2024

Electronic publication
