# Supplementary material for: Multi-omics characterization of esophageal squamous cell carcinoma identifies molecular subtypes and therapeutic targets
Source: JCI Insight. 2024 Apr 23;9(10):e171916. doi: 10.1172/jci.insight.171916 (PMC11141925; doi:10.1172/jci.insight.171916)
Supplement: Supplemental data [file jciinsight-9-171916-s071.pdf]

Supplemental Information

Multi-omics Characterization of Esophageal Squamous Cell Carcinoma Identifies Molecular Subtypes and Therapeutic Targets

Dengyun Zhao, Yaping Guo, Huifang Wei, Xuechao Jia, Yafei Zhi, Guiliang He, Wenna Nie, Limeng Huang, Penglei Wang, Kyle Vaughn Laster, Zhicai Liu, Jinwu Wang, Mee-Hyun Lee, Zigang Dong, and Kangdong Liu

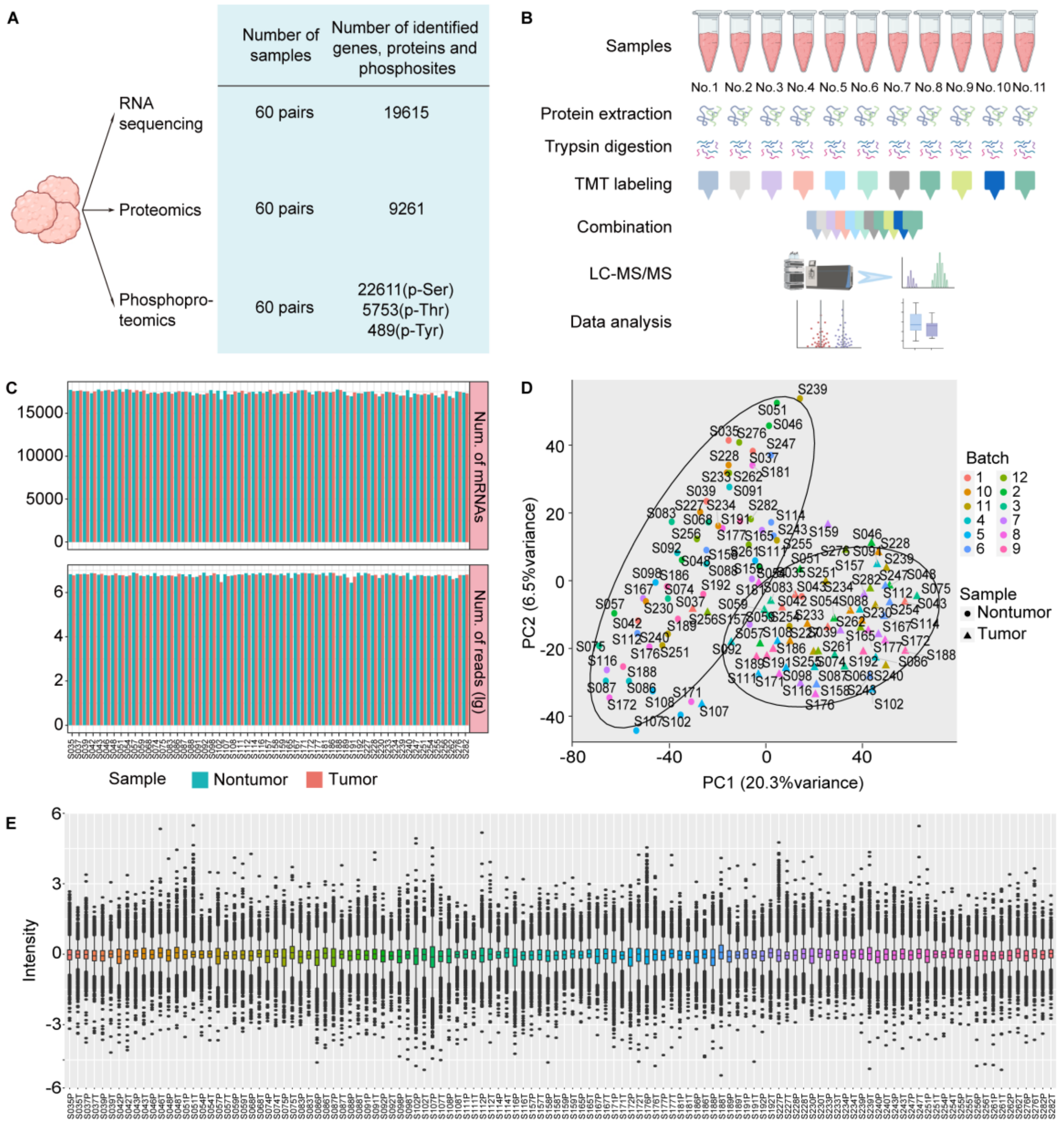

Supplemental Figure 1. Schematic of this study and quality control of the data.

(A) Schematic illustrating the multi-omics analyses performed on paired tumor and nontumor samples. The number of genes, proteins, and phosphosites identified in the indicated analysis pipelines are listed. (B) Workflow describing the proteomic and phosphoproteomic analyses utilizing TMT-11 plex. (C) Histograms depicting the distribution of mRNAs (top) and read counts (bottom) in each sample. Green bars represent nontumor tissue, while red bars represent tumor tissue. (D) Batch effect analysis among 12 TMT-11 plexes based on proteomics data. (E) Histograms demonstrating the relative abundance of identified proteins in all 60 paired samples.

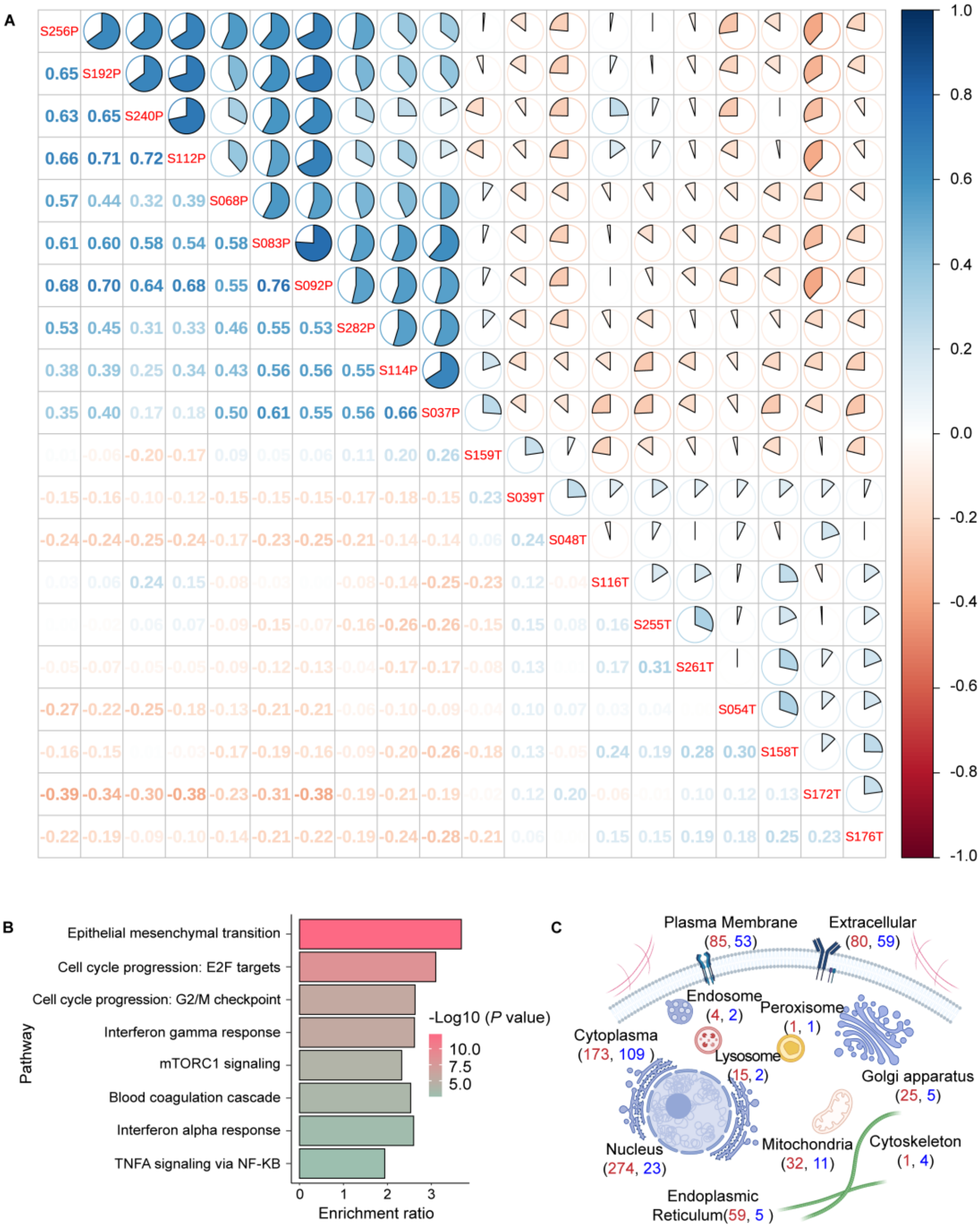

Supplemental Figure 2. Correlation coefficient analyses of samples and enrichment analysis of differentially expressed proteins.

(A) Correlation coefficient analysis of 10 tumors and 10 adjacent nontumor samples. (B) Histogram depicting the pathway enrichment of dysregulated proteins. (C) Schematic illustrating the subcellular localization of differential proteins. Red color represents upregulated proteins and blue color represents downregulated proteins.

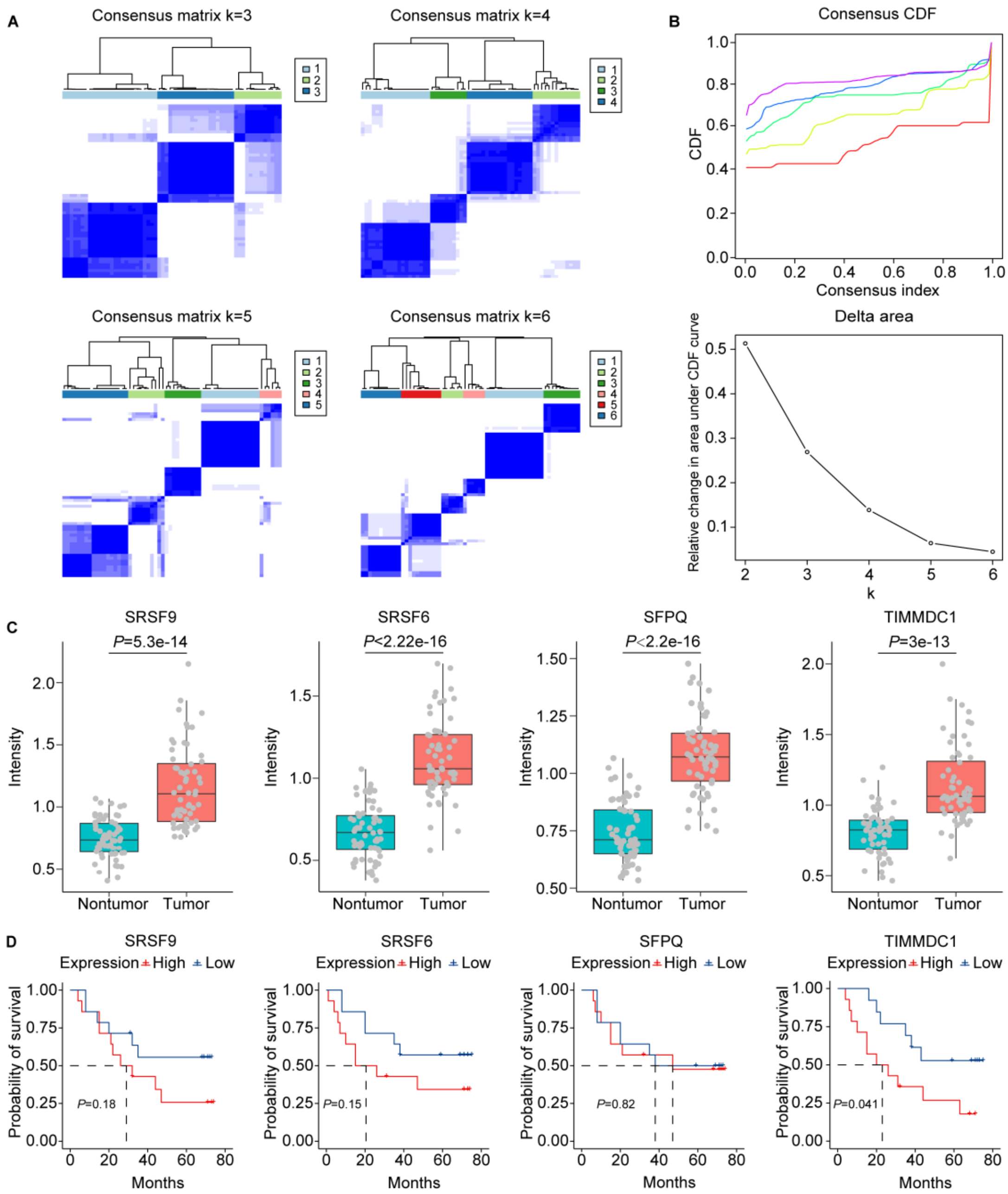

Supplemental Figure 3. Proteome consensus clustering results of all 60 paired ESCC samples.

(A) The optimal proteome clusters were visualized for these samples using 1,000 resampled data sets to explore clustering patterns within k=3 to k=6 range. (B) Cumulative distribution function (CDF) plots and Delta area plots were generated for consensus matrices at different values of k. (C) Histograms depict the protein expression levels of SRSF9, SRSF6, SFPQ, and TIMMDC1 in tumor and nontumor tissues, respectively. (D) Kaplan-Meier plots compare the survival probability of patients with high/low SRSF9, SRSF6, SFPQ, and TIMMDC1 expression, respectively.

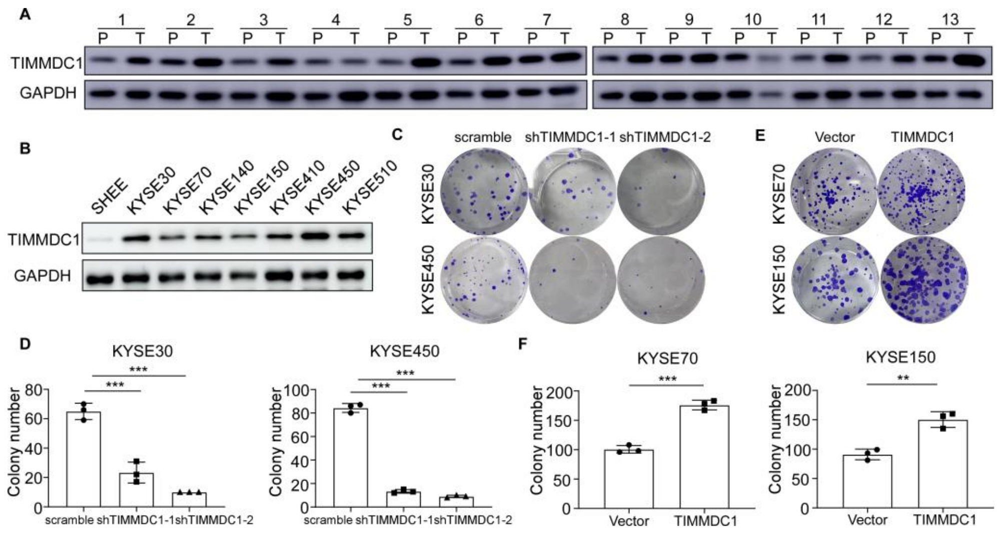

Supplemental Figure 4. Proliferation inhibitory effect of TIMMDC1 knockdown in ESCC.

(A) TIMMDC1 protein levels were assessed using Western blot analysis in paired tumor and adjacent nontumor tissues. P: Nontumor tissue; T: Tumor tissue. (B) Western blot analysis of TIMMDC1 expression levels in the normal esophageal epithelial cell line SHEE and 7 ESCC cell lines. (C and D) Colony formation ability was evaluated in KYSE30 and KYSE450 cell lines following infection with scramble and shTIMMDC1 lentivirus using a colony formation assay (*n* = 3 for each group). (E and F) Colony formation ability was assessed in KYSE70 and KYSE150 cells transfected with vector or TIMMDC1 plasmids using a colony formation assay (*n* = 3 for each group). In all statistical plots, data were expressed as the mean ± SD. 1-way ANOVA analysis (D) and 2-tailed student's *t* test (F) were used to determine statistical significance. ***P* < 0.01, ****P* < 0.001. Representative results from at least 3 independent biological replicates (C-F) are shown.

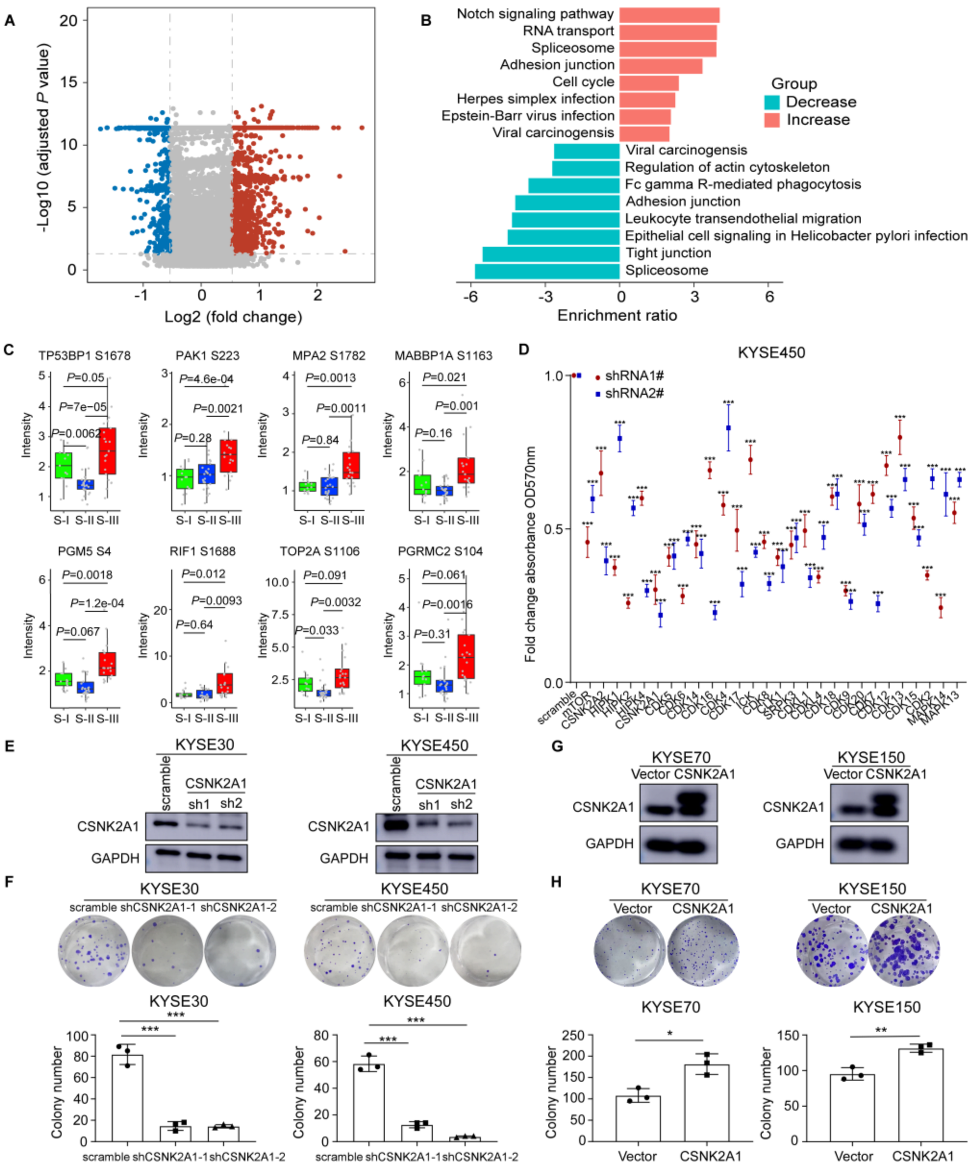

Supplemental Figure 5. Analysis of differential phosphoproteins and selection of CSNK2A1.

(A) The volcano plot illustrates the differential phosphoproteins observed in 60 patient samples. Red dots indicate upregulated phosphoproteins in tumor tissues compared to adjacent nontumor tissues, while blue dots represent downregulated phosphoproteins in tumor tissues compared to adjacent nontumor tissues. (B) The histogram displays the pathway enrichment of these differential phosphoproteins, where red bars indicate upregulated pathways in tumor tissues compared to adjacent nontumor tissues, and green bars represent downregulated pathways in tumor tissues compared to adjacent nontumor tissues. (C) The Histogram demonstrates the expression of representative phosphosites in S-I to S-III. (D) Cell proliferation at 72 hours was assessed using an MTT assay after knockdown of the indicated kinases in KYSE450 cell (n = 6 for each group). (E) CSNK2A1 knockdown efficiency was determined by Western blot analysis in KYSE30 and KYSE450 cells after infection with scramble and shCSNK2A lentivirus. (F) Colony formation ability was measured by using colony formation assay in KYSE30 and KYSE450 CSNK2A1 knockdown cells (n = 3 for each group). (G) CSNK2A1 overexpression efficiency was determined by Western blot analysis in KYSE70 and KYSE150 cells transfected with vector or CSNK2A1 overexpression plasmids. (H) Colony formation ability was measured by using colony formation assay in KYSE70 and KYSE150 CSNK2A1 overexpression cells (n = 3 for each group). In all statistical plots, data were expressed as the mean ± SD. 1-way ANOVA analysis (D and F) and 2-tailed Student's t test (H) were used to determine statistical significance. *P < 0.05, **P < 0.01, ***P <

0.001. Representative results from at least 3 independent biological replicates (D-H) are shown.

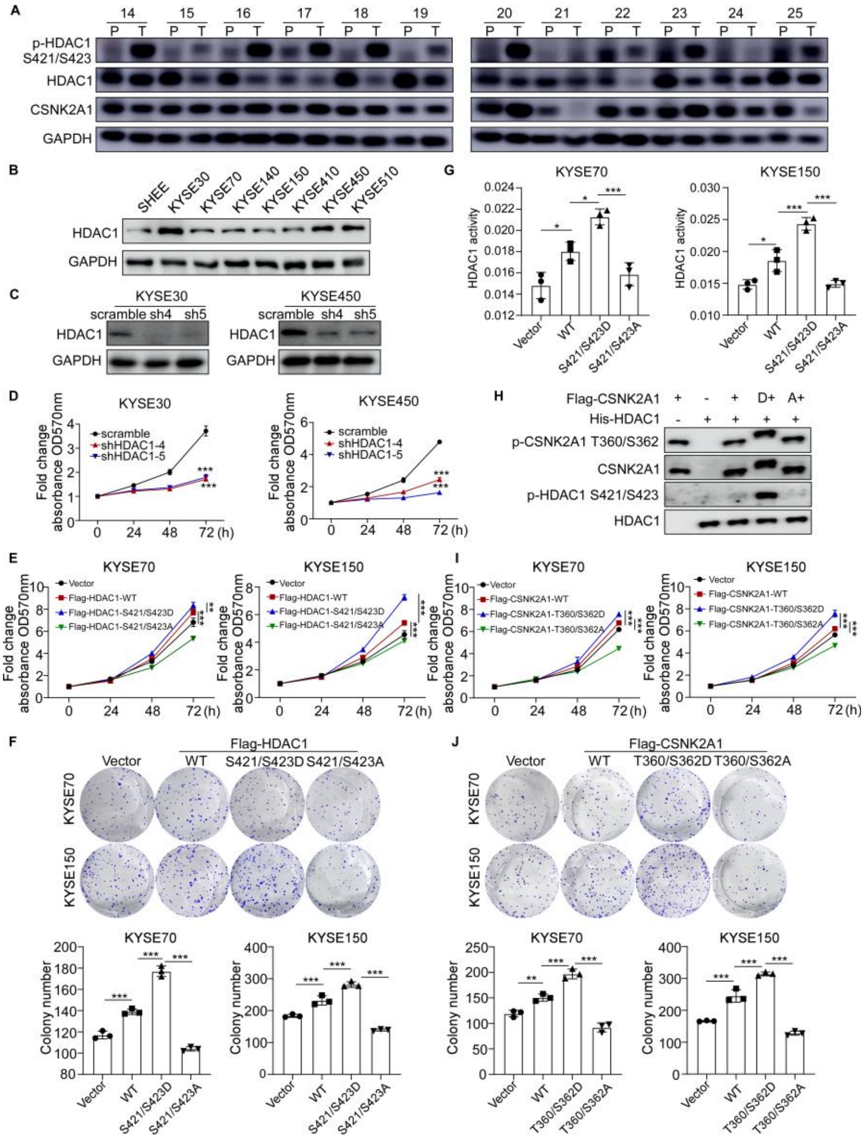

Supplemental Figure 6. Expression pattern and function of p-HDAC1 S421/S423 and p-CSNK2A1 T360/S362 in ESCC.

(A) The phosphorylation levels of CSNK2A1 substrates (p-HDAC1 S421/S423) were assessed by Western blot analysis using lysates derived from paired ESCC and adjacent nontumor samples. (B) Western blot analysis was performed to assess the protein levels of HDAC1 in the normal esophageal epithelial cell line SHEE and seven ESCC cell lines. (C) The effect of HDAC1 knockdown was confirmed by Western blotting in KYSE30 and KYSE 450 cells. (D) Cell proliferation of KYSE30 and KYSE450 HDAC1 knockdown cells was assessed using MTT assay (n = 6 for each group). (E-G) Flag-tagged HDAC1-WT, HDAC1-S421/S423D, and HDAC-S421/S423A plasmids were transfected into KYSE70 and KYSE 150 cells for subsequent analysis. Cell proliferation was assessed using the MTT assay (E) (n = 6 for each group), while cell colony formation ability was evaluated by using colony formation assay (n = 3 for each group) (F). Additionally, HDAC1 activity was determined through the HDAC1 activity assay (n = 3 for each group) (G). (H) Recombinant HDAC1 protein was incubated with active form of CSNK2A1 as well as phosphorylated forms CSNK2A1 T360/S362D and CSNK2A1 T360/S362A in a kinase reaction buffer for 30 minutes at 30 °C. Phosphorylation signals were detected using Western blot analysis. (I and J) Flag-tagged CSNK2A1-WT, CSNK2A1-T360/S362D, and CSNK2A1-T360/S362A plasmids were transfected into KYSE70 and KYSE 150 cells, followed by assessment of cell proliferation using MTT assay (n = 6 for each group) (I), while colony formation assay

(*n* = 3 for each group) was used to evaluate cell colony formation ability (J). In all statistical plots, data were expressed as the mean ± SD. 1-way ANOVA analysis (D-G, I, and J) was used to determine statistical significance. *$P < 0.05$, **$P < 0.05$, ***$P < 0.001$. Representative results from at least 3 independent biological replicates (C-J) are shown.

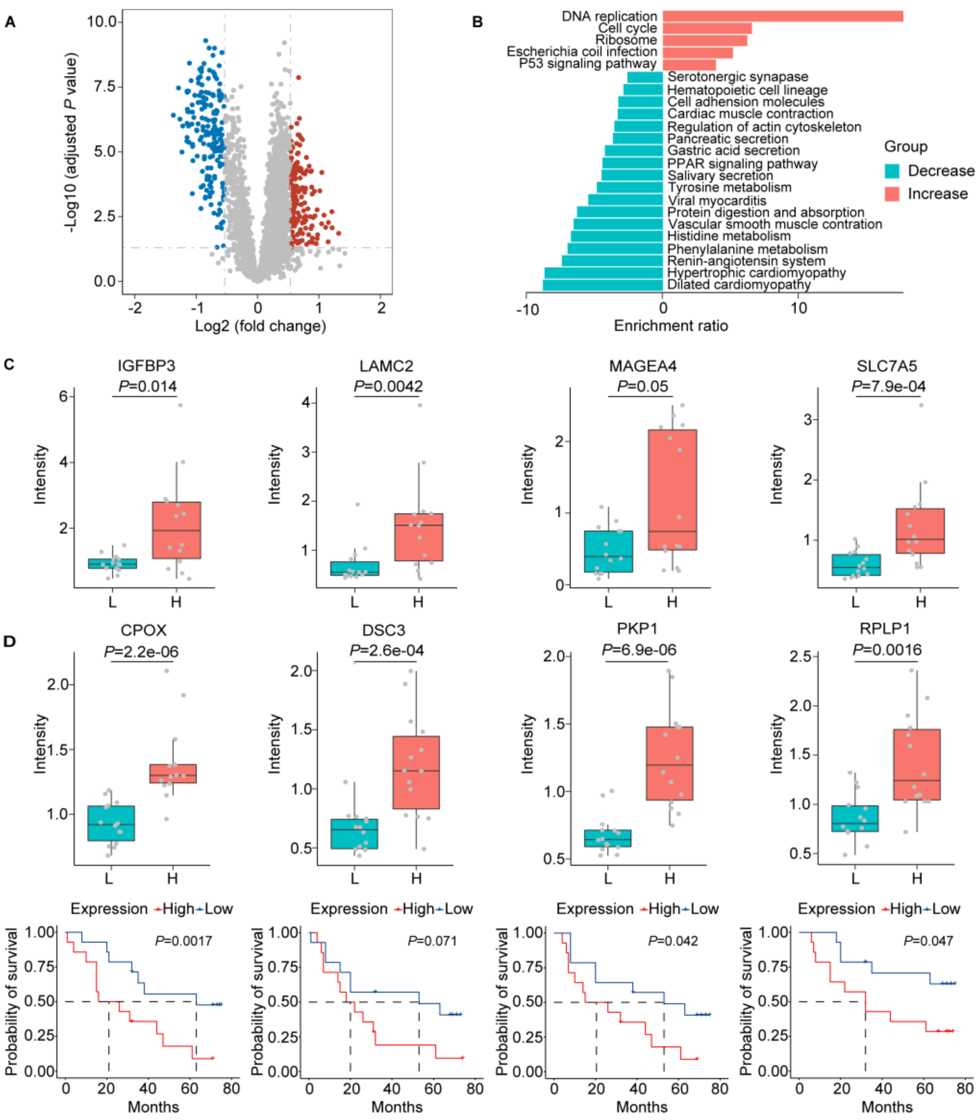

Supplemental Figure 7. Analysis of differential expression and pathway enrichment with high and low expression of CSNK2A1 in ESCC.

(A) The volcano plot displays the differentially expressed proteins between the CSNK2A1 high expression group and the CSNK2A1 low expression group. Red dots indicate upregulated proteins and blue dots represent downregulated proteins in the CSNK2A1 high expression group compared to the low expression group. Gray dots denote no significant difference. (B) The histogram illustrates pathway enrichment analysis of differentially expressed proteins in both groups. Red bars show upregulated pathways in the CSNK2A1 high expression group whereas green bars represent downregulated pathways. (C)The histogram shows the expression levels of representative differentially expressed protein reported in ESCC for both groups. H: CSNK2A1 high expression group; L: CSNK2A1 low expression group. (D) Histogram showing the expression and Kaplan-Meier analysis of representative proteins that were identified in ESCC in the CSNK2A1 high expression group compared to the CSNK2A1 low expression group.

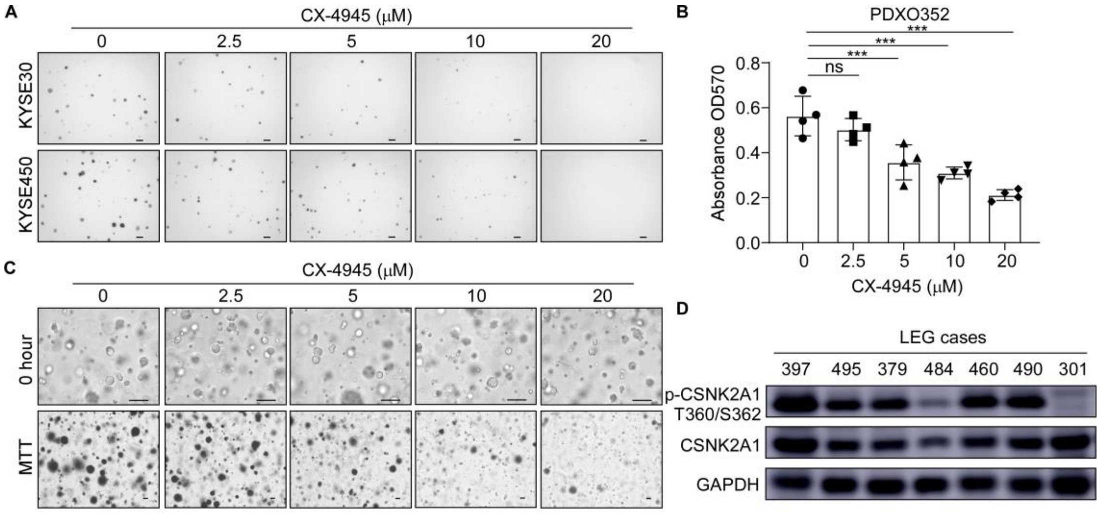

E

ID	Gender	Age(yrs)	Tumor	Source	TNM stage	Pathology grade	Differentiation	Lymphatic metastasis	Prior Chemo
LEG379	Female	64	ESCC	Primary	T3N2M0	IIIA	Middle	YES	NO
LEG397	Male	70	ESCC	Primary	T3N2M0	IIIB	Middle	YES	NO
LEG460	Male	71	ESCC	Primary	T3N1M0	IIIB	Middle	YES	NO
LEG244	Male	66	ESCC	Primary	T3N0M0	IIB	Middle	NO	NO

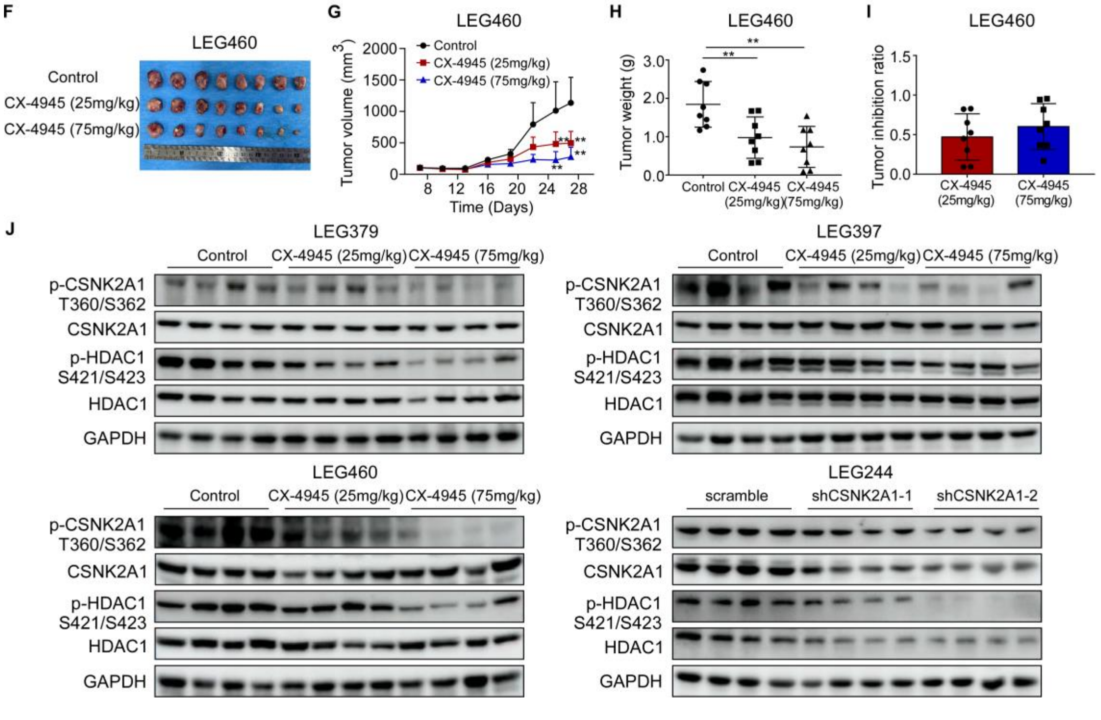

Supplemental Figure 8. Effect of CX-4945 on cell and PDX models.

(A) The effect of CX-4945 treatment on cell growth in KYSE30 and KYSE450 cells was evaluated using the soft agar assay. Scale bar: 200μm. (B and C) Organoid growth was assessed after exposure to CX-4945 at concentrations ranging from 0 μM to 20 μM (n = 4 for each group). Scale bar: 100μm. (D) The levels of p-CSNK2A1 T360/S362 in PDX tumor samples were determined by Western blot analysis. (E) Clinical information for cases LEG379, LEG397, LEG460, and LEG244. (F) LEG460 tumors were excised from mice treated with CX-4945 (n = 8 for each group). (G-I) The PDX tumor growth curve (G), tumor weights (H), and tumor inhibition ratios (I) are presented for the LEG460 case treated with CX-4945. (J) The levels of both total and phosphorylated CSNK2A1 (p-CSNK2A1 T360/S362) and HDAC1 (p-HDAC1 S421/S423) were assessed in PDX cases of LEG379, LEG397, LEG460, and LEG244. 1-way

ANOVA analysis (B, G, and H) was used to determine statistical significance. $^{**}P < 0.01$, $^{***}P < 0.001$. Representative results from at least 3 independent biological replicates (A-C) are shown.

SUPPLEMENTARY METHODS

Total RNA extract

The Trizol method was employed for the extraction of total RNA from tissues. Approximately 50-100 mg of tissue from each sample was finely grinded in liquid nitrogen using a mortar and pestle. Subsequently, Trizol (Invitrogen, 15596026) was added to the samples which were then homogenized and incubated at RT for 5 minutes. The mixture underwent centrifugation at 12,000×g for 5 minutes at 4°C. Afterward, supernatants were collected into tubes with addition of chloroform. After vortexing for 15 seconds, the mixtures were subjected to further centrifugation at 12,000×g for 10 minutes at 4°C. The aqueous phase was aspirated and transferred into a tube. Isopropyl alcohol was then added, followed by centrifugation of the mixture at 12,000×g for 10 minutes at 4°C. The supernatant was then discarded and the RNA pellets were washed twice with 1 mL of 75% ethanol, vortexed, and centrifuged at 7,500×g for 5 minutes at 4°C. The supernatant was then removed, and the tubes were air dried for 5 minutes. DEPC water was used to resuspend the RNA pellets. RNA concentration was quantified using a NanoDrop (Thermo Scientific).

RNA library construct

The mRNA samples were enriched using magnetic beads, followed by removal of rRNA with RNase H. Subsequently, the purified mRNA was fragmented into small pieces. First-strand cDNA and second-strand cDNA were generated using PCR methods. Afterward, the PCR reaction product was purified using magnetic beads. The purified cDNA fragments were then combined with A-Tailing Mix and RNA Index Adapters amplified products. Further purification of the cDNA fragments was performed using AMPure XP Beads (Beckman, A63881). The

library quality was validated using Agilent Technologies 2100 Bioanalyzer. Finally, the double-stranded PCR products obtained from the aforementioned methods underwent heating, denaturation, and circularization processes to form single-strand circular DNA as the final library. Three samples (S176T, S243T, and S261T) were excluded due to inadequate quality.

Total RNA sequencing and Quantification of gene expression

The phi29 DNA polymerase was utilized for the amplification of the final library and construction of a DNA nanoball, which contained more than 300 copies of each molecule. Subsequently, the DNBs were loaded into a patterned nanoarray, and single end 50 base reads were generated using the BGISEQ500 platform. To ensure high quality results, low quality reads, joint contamination, and sequences with high unknown base N content were excluded before the data analysis. The clean reads were then aligned against the NCBI reference genome version GCF_000001405.39_GRCH38.p13 using HISAT.

The overall average mapping rate per sample was determined to be 89.2%, and the gene expression quantification was performed on mapped reads using the ‘classic-tpm’ library normalization method based on Ensemble transcripts annotation. Genes exhibiting stable expression are defined as those with transcripts per kilobase of exon model per million mapped reads (TPM) markers reads > 1 in at least one sample. Differential gene expressions between tumor and adjacent nontumor tissues in 60 pairs were assessed using Wilcoxon rank-sum test implemented in R software, while multiple testing correlation was conducted using the Benjamin-Hochberg procedure. Differentially expressed genes were identified based on fold change expressed as Log2 (ratio of average TPM in tumor vs nontumor) > 1 or < -1 and Benjamini-Hochberg adjusted $P < 0.05$.

Protein extraction

The tumor and adjacent nontumor tissues, weighing approximately 50-100 mg each, were finely powdered in liquid nitrogen using a mortar and pestle. Subsequently, the tissue powders were mixed with five volumes of lysis buffer (8 M urea, 1% Protease Inhibitor Cocktail, and 1% Phosphatase Inhibitor Cocktail). Ultrasonication was performed three times on ice to disrupt the tissues. Following this step, the lysates were centrifugated at 12,000×g at 4°C for 15 minutes to obtain clarified supernatant. Protein concentrations in the supernatant were determined using a BCA kit.

Trypsin Digestion

The protein lysate was supplemented with 5 mM dithiothreitol (Sigma-Aldrich, D9760) and incubated at 56°C for 30 minutes to facilitate protein reduction. Subsequently, 11 mM iodoacetamide (Sigma-Aldrich, I1149) was added and the mixture was incubated in the dark at RT for 15 minutes to promote protein alkylation. To maintain a urea (Sigma-Aldrich, U5378) concentration below 2 M, 100 mM TEAB (Sigma-Aldrich, T7408) was introduced into the lysate. Trypsin (Promega, V5111) digestion was initiated by adding trypsin at a mass ratio of 1:50 (trypsin: protein), followed by overnight incubation at 37°C. The next day, additional trypsin was added at a mass ratio of 1:100 (trypsin: protein) and digestion continued for 4 hours.

TMT labeling

The Strata-X C18 Solid-phase extraction column (Phenomenex, 8B-S038-UBJ) was employed for desalting tryptic peptides, followed by drying using a Speed-Vac. Subsequently, the dried tryptic peptides were reconstituted with 0.5 M TEAB solution and labeled according to the

manufacturer's instructions using the TMT kit (Thermo Scientific). Briefly, acetonitrile (Fisher Chemical, A955) was used to dissolve the thawed TMT-labeled reagents which were then mixed with the peptide. The resulting mixture underwent incubation at RT for 2 hours, followed by desalting and subsequent freeze-drying under vacuum conditions. In total, this study generated 12 TMT11 plexes comprising 5 patient samples each along with an internal reference sample. Equal amounts of peptide from both tumor and nontumor (a total of 120 samples from 60 patients) were combined into the internal reference sample.

HPLC Fractionation

The peptides were separated using a high pH reverse-phase HPLC system (Agilent Technologies) equipped with an Extend-C18 column (Agilent Technologies, 770995-902). Peptides were eluted with a gradient of acetonitrile (pH 9.0) over 60 minutes at a flow rate of 0.8 mL/min and collected as one fraction per minute. A total of 60 subfractions were obtained and subsequently consolidated into 18 fractions. The combined fractions were freeze-dried under a vacuum for further processing. For phosphorylation tryptic peptides, an initial separation into 60 fractions was performed, followed by consolidation into eight components.

Phosphopeptide Enrichment Using IMAC

The peptide mixtures were dissolved in a loading buffer (50% acetonitrile/6% trifluoroacetic acid). Subsequently, the dissolved peptide mixtures were incubated with IMAC microspheres under vibration conditions. After centrifugation, the supernatant was discarded to collect the enriched phosphopeptides. To remove nonspecifically absorbed peptides, the IMAC microspheres with enriched phosphopeptides were washed using a solution containing 50% acetonitrile/6% trifluoroacetic acid, followed by further removal using a solution of 30%

acetonitrile/0.1% trifluoroacetic acid. An elution buffer containing 10% NH_4OH was used to elute the enriched phosphopeptides from the IMAC microspheres. The supernatant containing phosphopeptides was collected and subjected to lyophilization for salt removal using C18 ZipTips according to manufacturer's instructions. Finally, the supernatant was lyophilized again for LC-MS/MS analysis.

LC-MS/MS Analysis

The lyophilized peptides were dissolved in a 0.1% (V/V) formic acid aqueous solution (Fluka, 94318) and subsequently separated using an Easy nLC-1000 ULTRA-high performance liquid system (Thermo Fisher Scientific). For proteomic analysis, the peptides were separated with the solvent B (0.1% formic acid in 90% acetonitrile) with a gradient ranging from 6% to 23% over 45 minutes, followed by an increase to 34% over 10 minutes, further climbing to 70% within 2 minutes, and then held at this concentration for the final 3 minutes, all at a constant flow rate of 700 nL/min. For phosphoproteomic analysis, the peptides underwent separation with solvent B (0.1% formic acid in 90% acetonitrile) with a gradient ranging from 4% to 20% over 46 minutes, followed by an increase to 30% over 8 minutes, further climbing to 80% within 3 minutes, and then held at this concentration for the final 3 minutes, all at a constant flow rate of 300 nL/min. Subsequently, the peptides were subjected to an NSI source followed by a Q ExactiveTM Plus mass spectrometer (Thermo Fisher Scientific). The electrospray voltage applied was 2.0 kV. The peptides were analyzed using the Orbitrap detector with a scan pan ranging from 350 to 1800 m/z. NEC was set as 28 to select the precursors for MS/MS. The dynamic exclusion was set to 15 seconds.

Data Normalization

The MaxQuant (v1.6.1.0) search engine was employed to identify peptides and phosphopeptides from proteomic and phosphoproteomic MS/MS spectra, respectively, with consistent parameters being used in each reference database for standard database search. Intensity values calculated by the MaxQuant software were adjusted based on mixed samples, followed by normalization using the median centering method, across all proteins or phosphosites to account for sample loading variations, and Log2 transformed if necessary.

Missing Value Imputation

The missing values were imputed using the normal distribution imputation method implemented in Perseus 1.6.14. Before missing value imputation, only proteins quantified in more than 50% of samples were retained to ensure sufficient data availability for accurate imputation. A distribution model with a downshift of 1.8 standard deviations (SD) and a width of 0.3 SD was automatically applied for the missing value estimation process. For algorithms that were not suitable for handling missing values, this procedure was performed before the statistical analysis.

Batch effect and data quality analysis for proteomic data

The unsupervised principal component analysis (PCA) was performed to assess the impact of TMT multiplexes on the proteomic data, aiming to assess any potential batch effect. The leading PCs derived from the proteomic data successfully distinguished tumor samples from nontumor samples. The samples in the same batch did not cluster together, suggesting that no obvious batch effect among the 12 TMT batches.

Differential protein and phosphoproteins analysis

Upregulation or downregulation proteins are defined as those showing significant differential

expression in tumors compared to their corresponding nontumors (T/P > 1.45 or < 1/1.45, Benjamini-Hochberg adjusted $P < 0.05$).

Integrative Network Analysis

The integrative network analysis was conducted using RNA-seq, proteomic, and phosphoprotemic data. Subsequently, an integrative molecular network diagram was constructed with data of differential mRNA, proteins, and phosphoproteins. These data were selected based on the following criteria: For differential mRNA, a threshold of Log2 FC > 1 or Log2 FC < -1 with a P value< 0.05 was applied; for differential proteins and phosphoproteins, a threshold of FC > 1.45, or FC < 1/1.45 with a P value< 0.05 was applied. Pathway enrichment analysis was then performed on the selected molecules revealing increased protein enrichment in cell cycle, WNT signaling, MAPK signaling, tight junction, ECM, cholesterol metabolism, and spliceosome pathways. Mapping of the differential mRNA/proteins/phosphoproteins to each identified pathway in KEGG was performed, yielding fold change values and corresponding P values for each analyzed molecule. The information regarding the subtype associated with each protein was also provided.

MTT

The KYSE30 cells were seeded at a density of $1×10^3$ cells/well in four 96-well plates. Similarly, the KYSE450 cells were seeded at a density of $2×10^3$ cells/well in four 96-well plates. Cells were incubated for different times (0, 24, 48, and 72 hours). At each time point, MTT solution (Solarbio, M8180) was added to the wells and incubated for 2 hours at 37°C. Subsequently, the media was replaced with DMSO (100 µL), followed by gentle agitation to dissolve formazan crystals. The absorbance was measured at OD570 using a microplate reader to

calculate the rate of proliferation based on OD570 values obtained at different time points. For the CX-4945 treated cells, concentrations of CX-4945 used were 0, 2.5, 5, 10, and 20 μM, respectively.

Colony formation assay

For the colony formation assay, cells were seeded at a density of 200 cells/well in 6-well plates. Subsequently, they were incubated in a CO_2 incubator for 9 to 11 days, followed by aspiration of the culture medium and fixation with methanol. After cell washing with PBS, crystal violet staining was performed to visualize the colonies under an inverted microscope. Colony quantification was carried out using Image-Pro Plus software (v.6.0) program (Media Cybernetics, Rockville, MD). The number of colonies was then calculated.

Soft agar assay

The Cells (8×10^3cells/well) were seeded into the bottom medium in 6-well plates and cultured for 1 to 2 weeks. The number of colonies was quantified using the Image-Pro Plus software program, and the rate of colony growth was calculated. For the CX-4945 treated cells, different concentrations of CX-4945 (0, 2.5, 5, 10, and 20 μM) were used respectively.
