# Supplementary material for: Multi-omics characterization of esophageal squamous cell carcinoma identifies molecular subtypes and therapeutic targets
Source: JCI Insight. 2024 Apr 23;9(10):e171916. doi: 10.1172/jci.insight.171916 (PMC11141925; doi:10.1172/jci.insight.171916)

Full unedited gel for Figure 4F

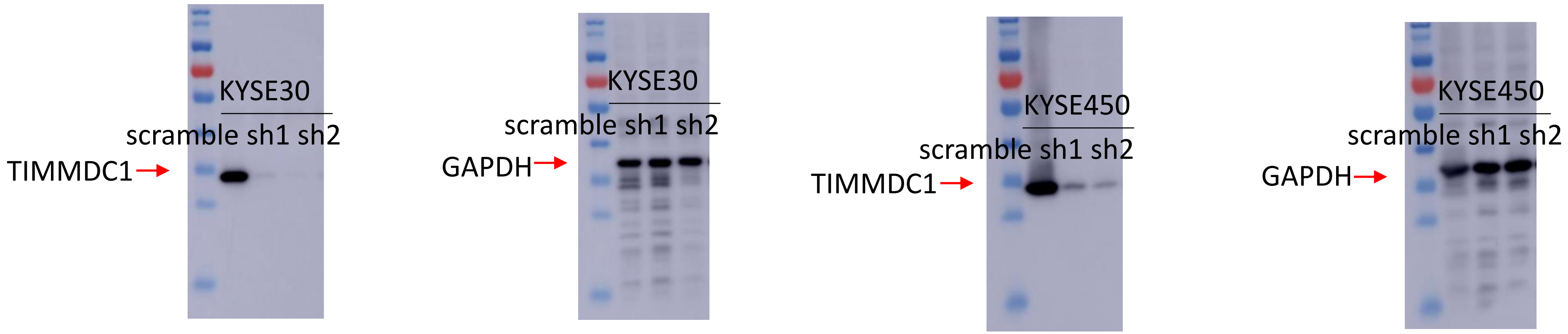

Full unedited gel for Figure 4I

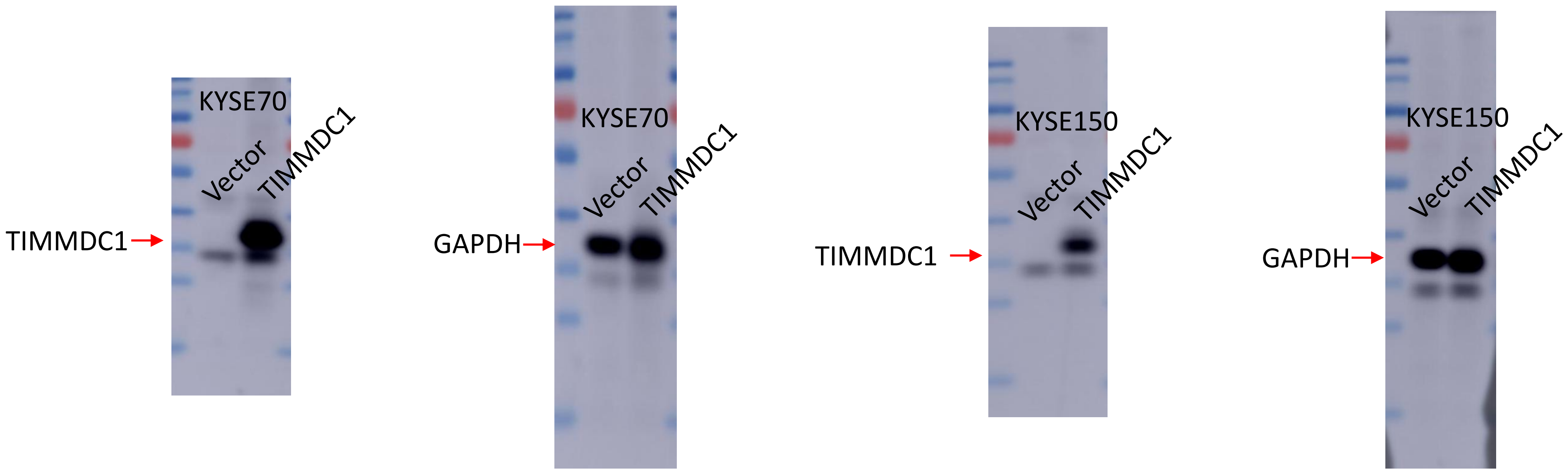

Full unedited gel for Supplemental Figure 4A

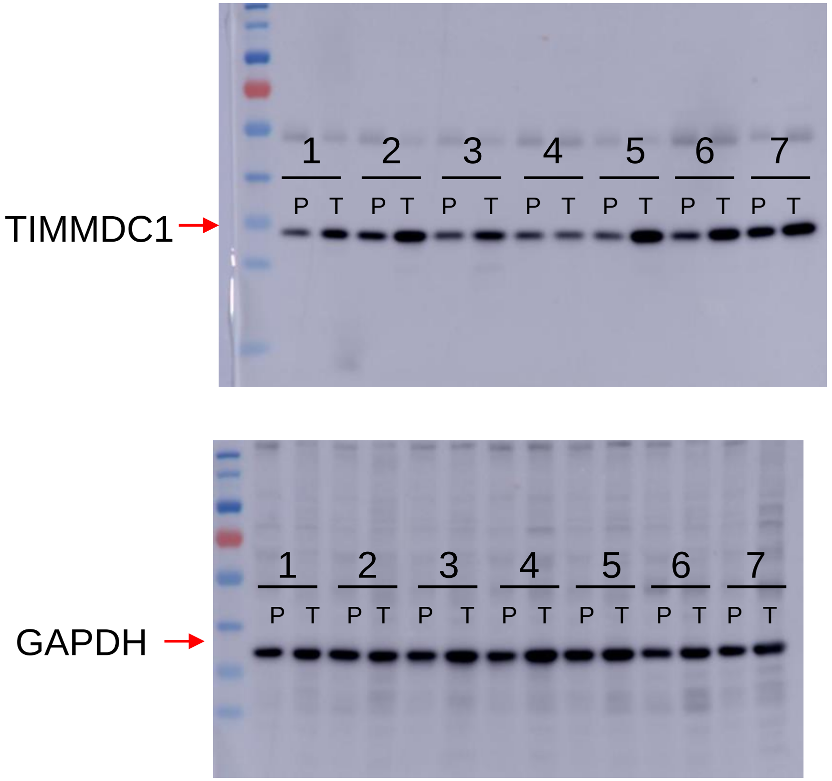

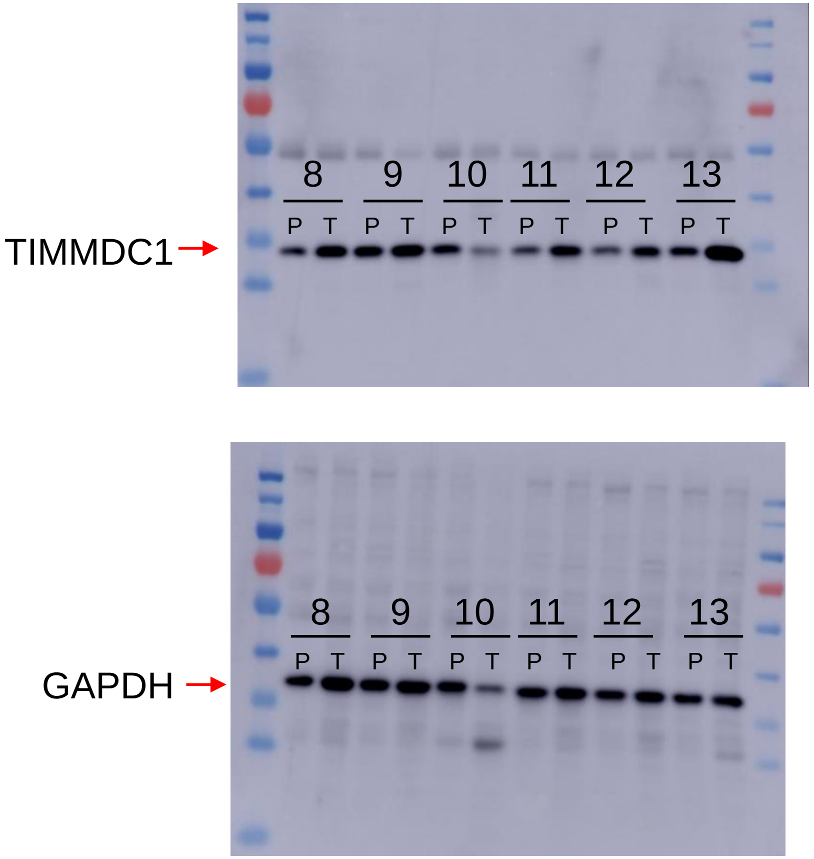

Full unedited gel for Supplemental Figure 4B

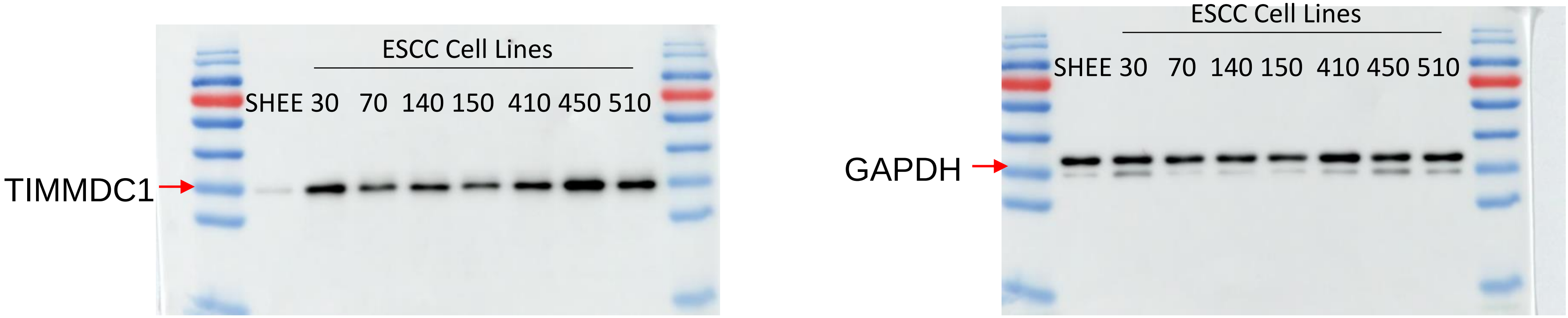

Full unedited gel for Supplemental Figure 5E

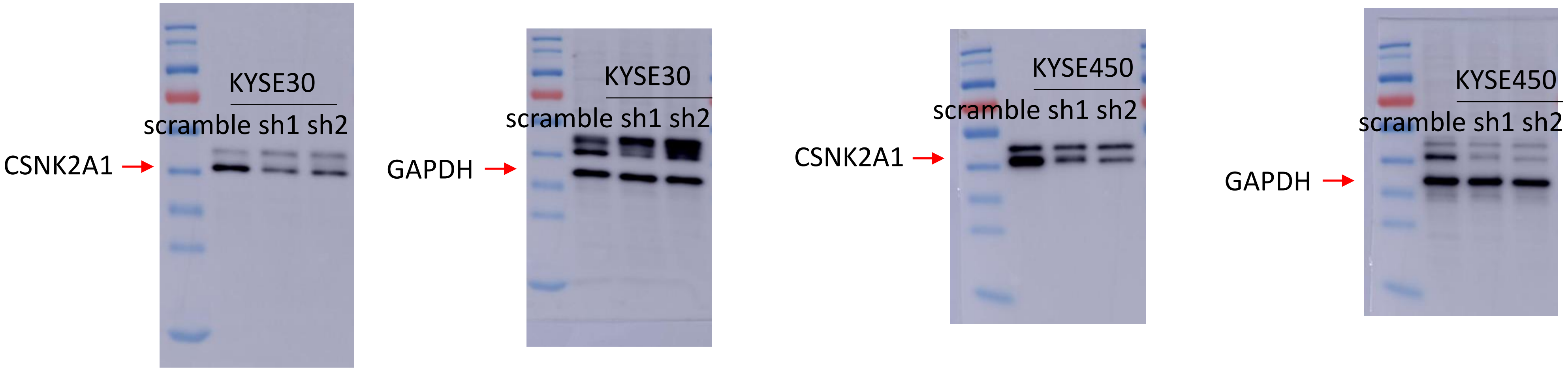

Full unedited gel for Supplemental Figure 5G

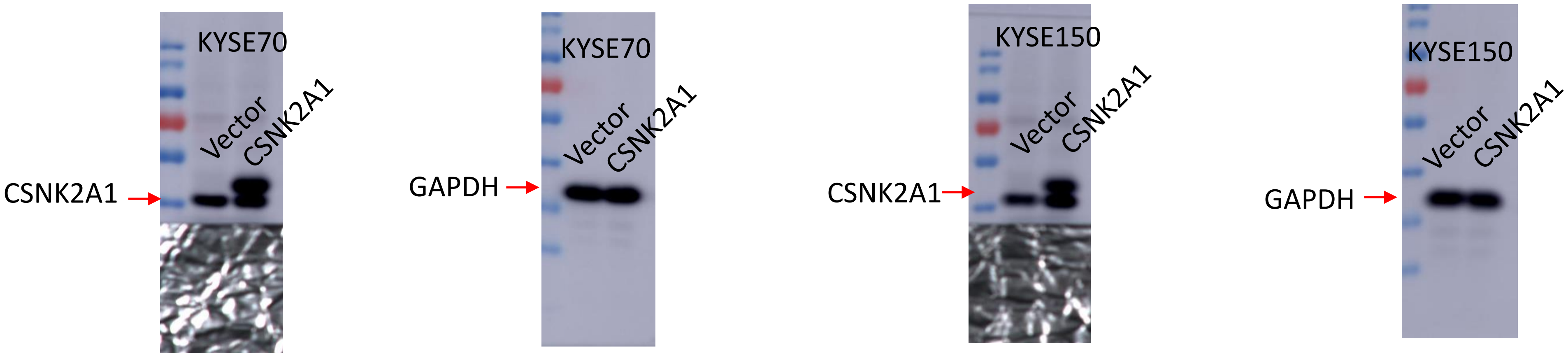

Full unedited gel for Supplemental Figure 6A

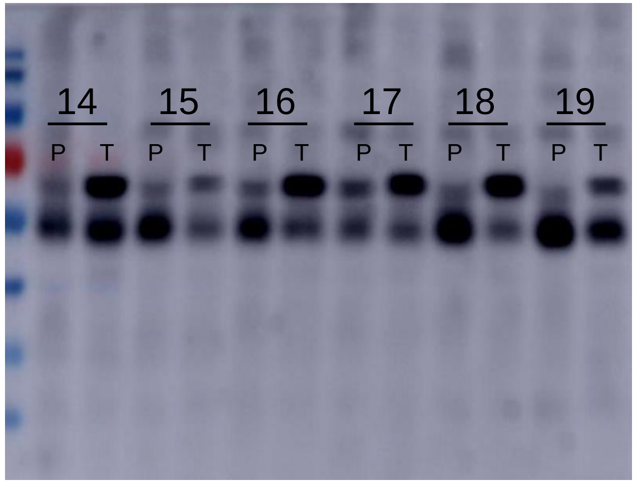

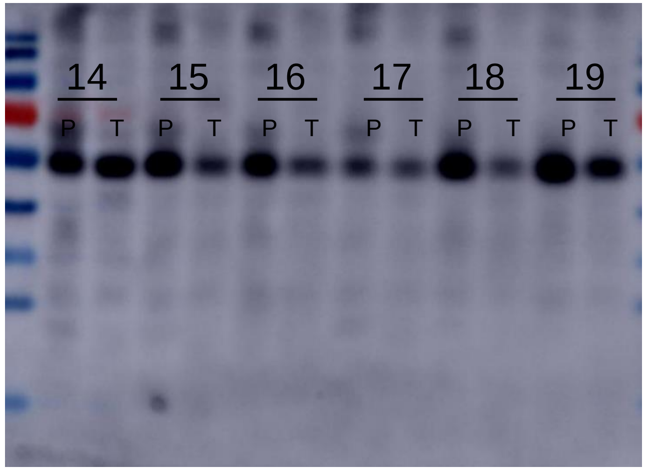

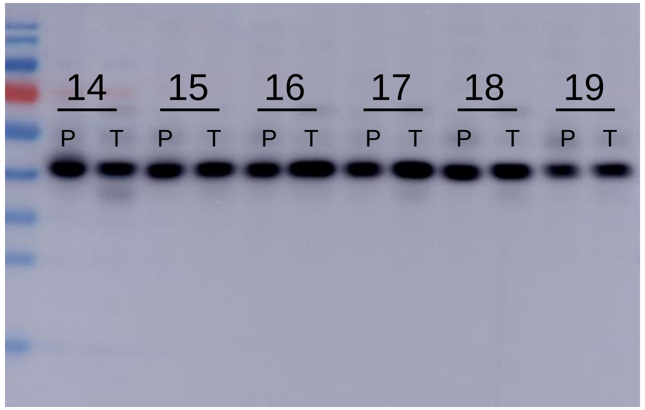

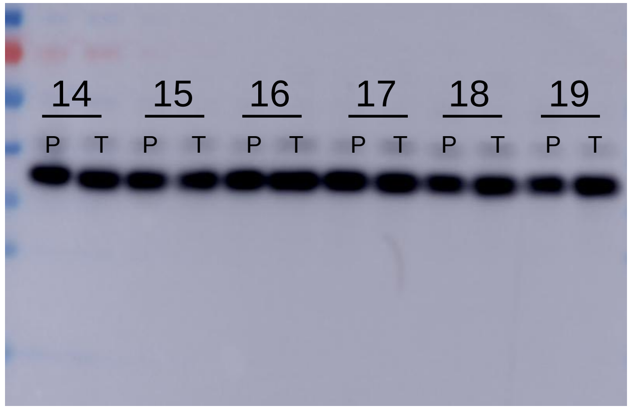

Full unedited gel for Supplemental Figure 6A

p-HDAC1
S421/S423

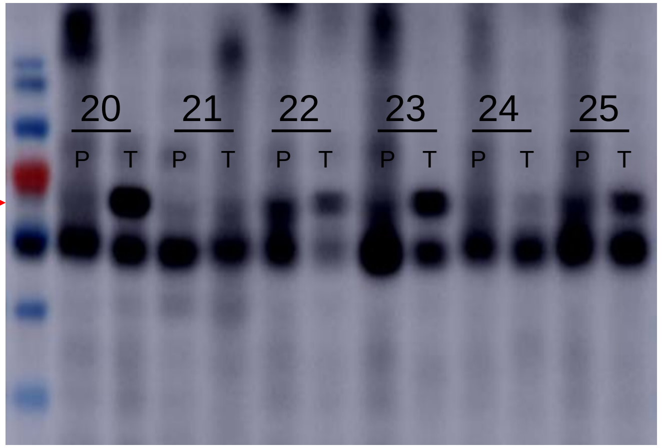

HDAC1

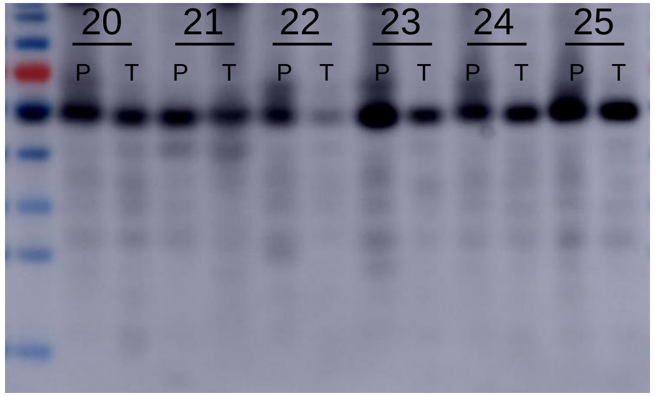

CSNK2A1

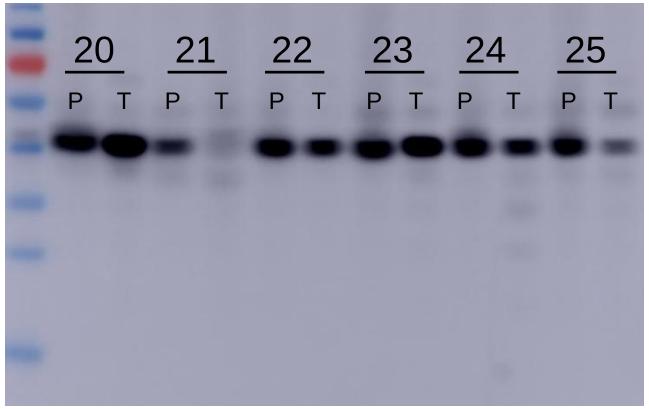

GAPDH

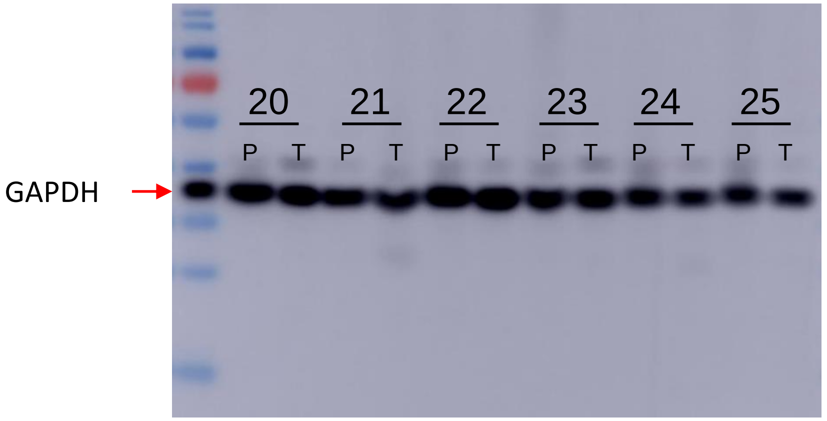

Full unedited gel for Supplemental Figure 6B

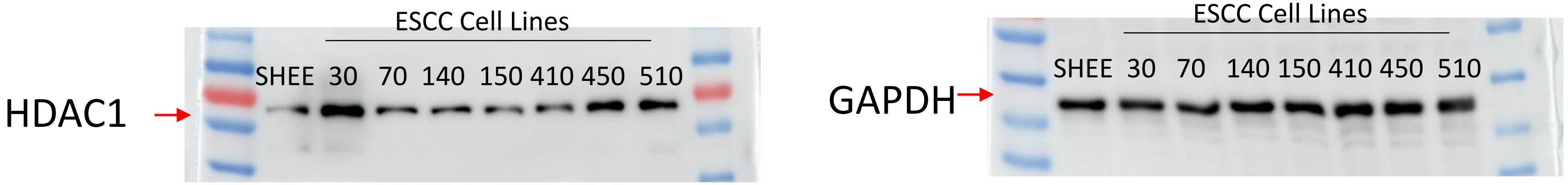

Full unedited gel for Supplemental Figure 6C

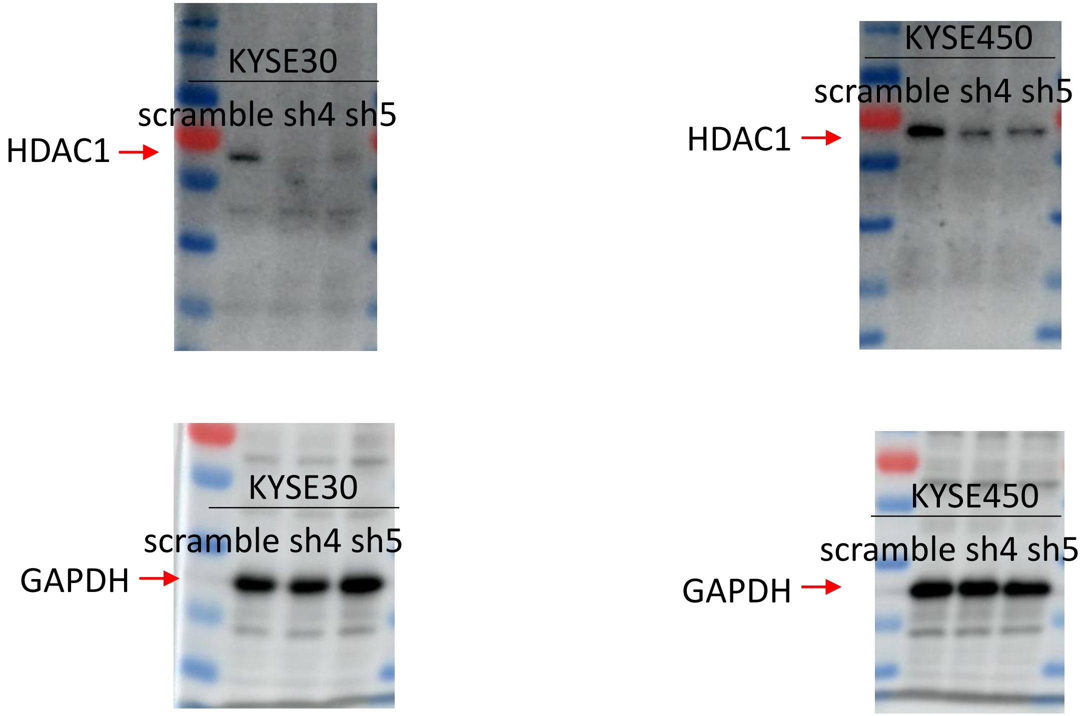

Full unedited gel for Supplemental Figure 6H

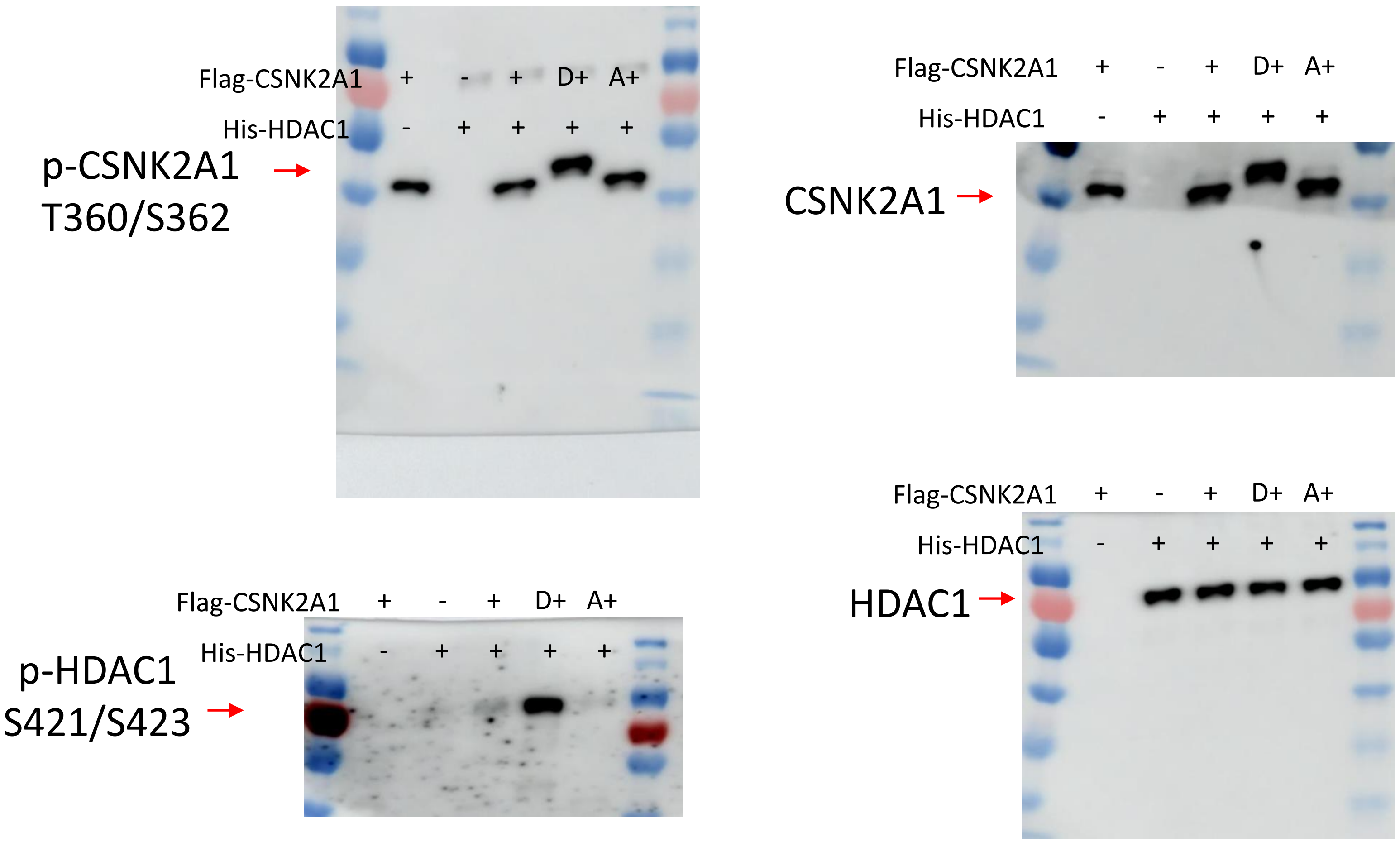

Full unedited gel for Figure 6D

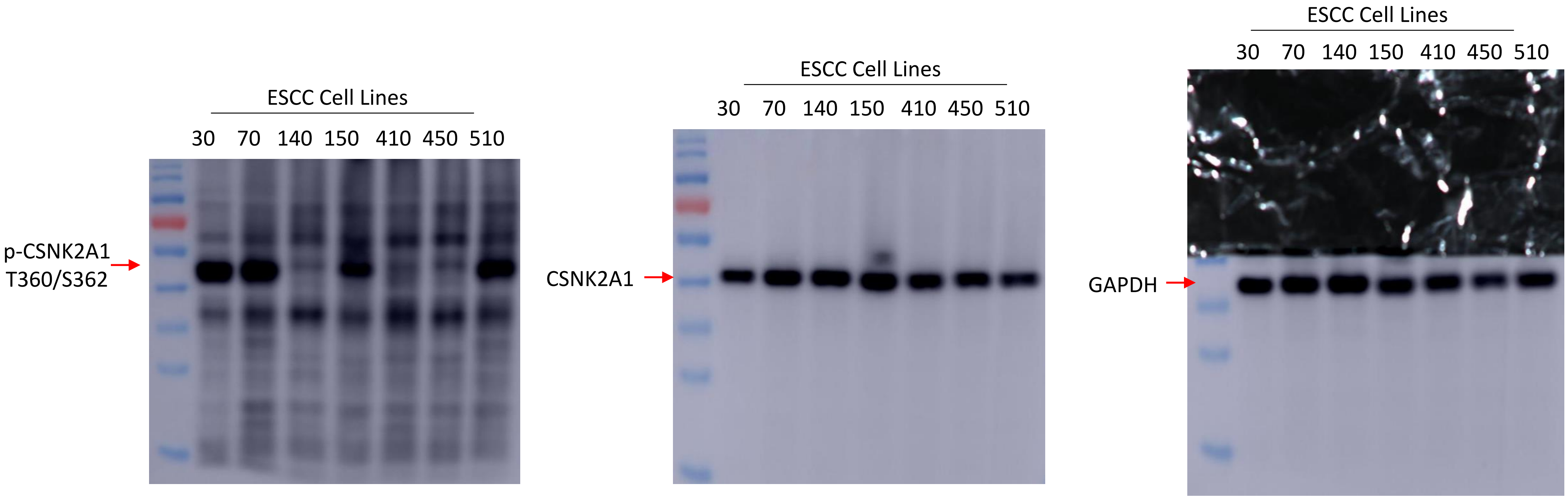

Full unedited gel for Supplemental Figure 8D

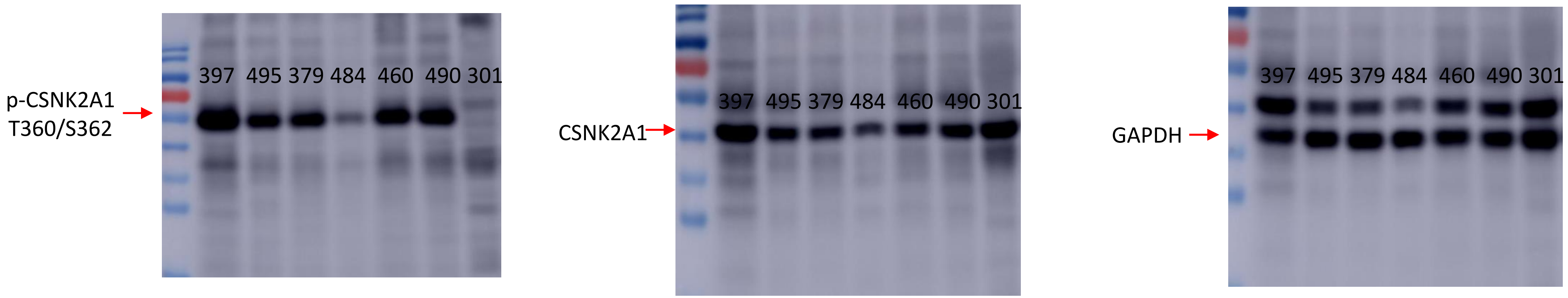

Full unedited gel for Supplemental Figure 8J

LEG379

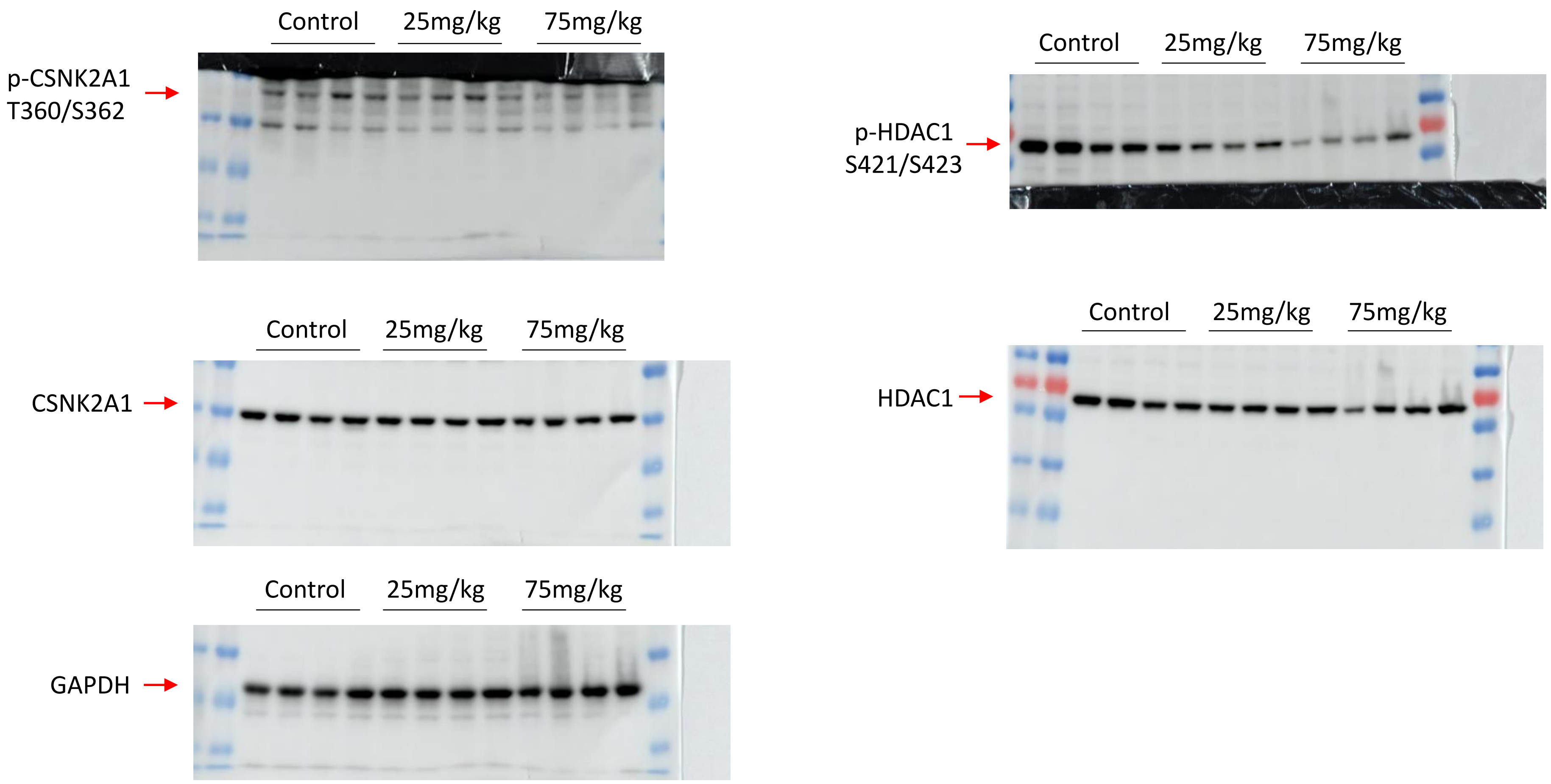

Full unedited gel for Supplemental Figure 8J

LEG397

Control 25mg/kg 75mg/kg

p-CSNK2A1 T360/S362 →

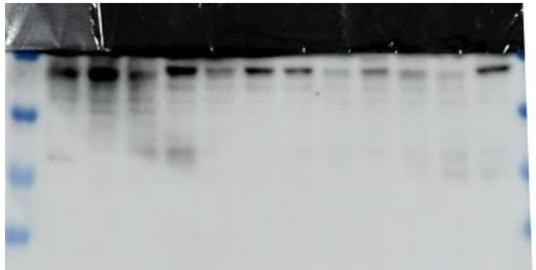

Control 25mg/kg 75mg/kg

p-HDAC1 S421/S423 →

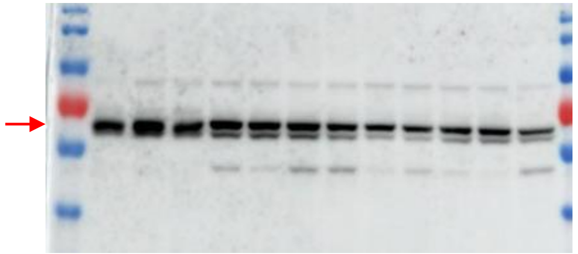

Control 25mg/kg 75mg/kg

CSNK2A1 →

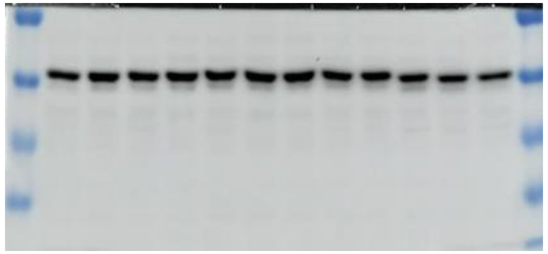

Control 25mg/kg 75mg/kg

HDAC1 →

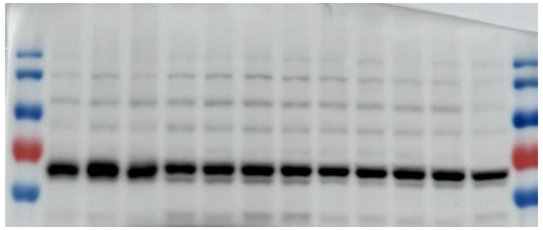

Control 25mg/kg 75mg/kg

GAPDH →

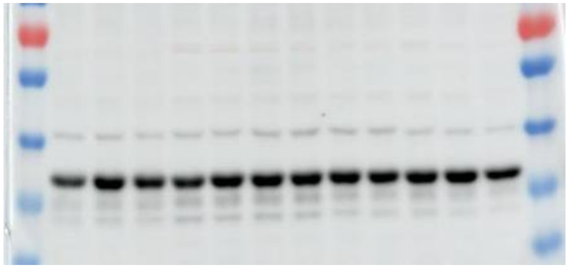

Full unedited gel for Supplemental Figure S8J

LEG460

Control 25mg/kg 75mg/kg

p-CSNK2A1 T360/S362

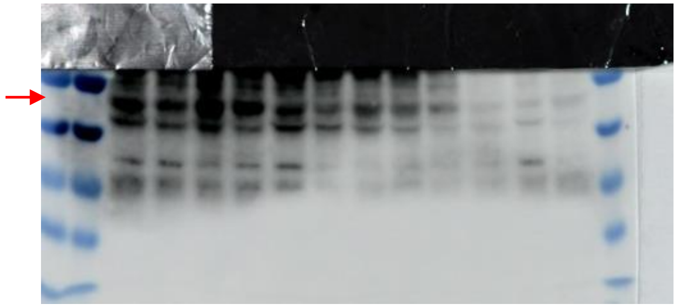

Control 25mg/kg 75mg/kg

p-HDAC1 S421/S423

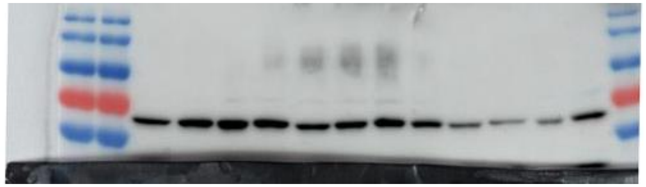

Control 25mg/kg 75mg/kg

CSNK2A1

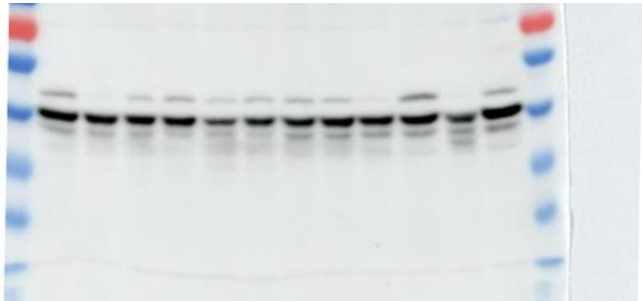

Control 25mg/kg 75mg/kg

HDAC1

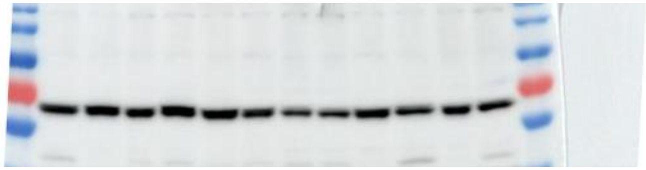

Control 25mg/kg 75mg/kg

GAPDH

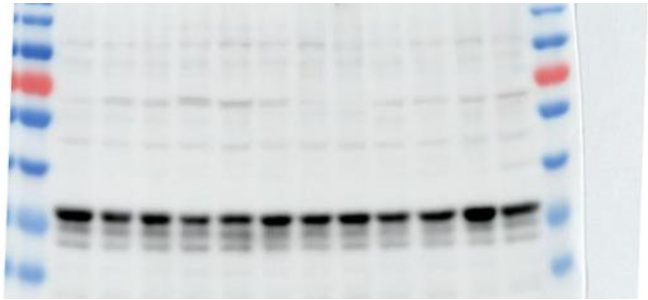

Full unedited gel for Supplemental Figure 8J

LEG244

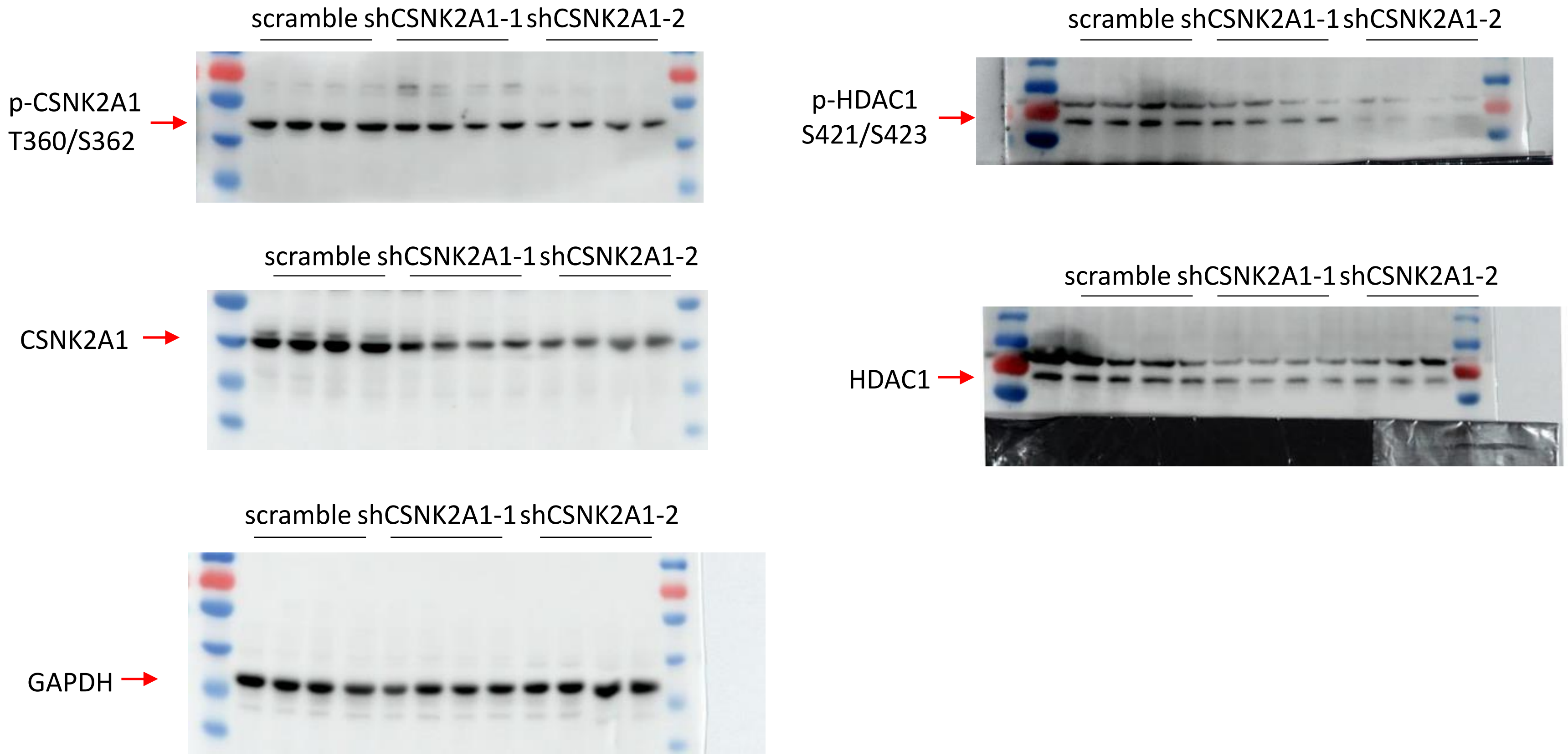

Supplement: Unedited blot and gel images [file jciinsight-9-171916-s072.pdf]
